# Extrapolative prediction using physically-based QSAR

**DOI:** 10.1007/s10822-016-9896-1

**Published:** 2016-02-10

**Authors:** Ann E. Cleves, Ajay N. Jain

**Affiliations:** Helen Diller Family Comprehensive Cancer Center, University of California, San Francisco, CA USA; Department of Bioengineering and Therapeutic Sciences, University of California, San Francisco, CA USA

**Keywords:** QSAR, QMOD, Surflex, Extrapolation, Binding mode prediction, Affinity prediction

## Abstract

Surflex-QMOD integrates chemical structure and activity data to produce physically-realistic models for binding affinity prediction . Here, we apply QMOD to a 3D-QSAR benchmark dataset and show broad applicability to a diverse set of targets. Testing new ligands within the QMOD model employs automated flexible molecular alignment, with the model itself defining the optimal pose for each ligand. QMOD performance was compared to that of four approaches that depended on manual alignments (CoMFA, two variations of CoMSIA, and CMF). QMOD showed comparable performance to the other methods on a challenging, but structurally limited, test set. The QMOD models were also applied to test a large and structurally diverse dataset of ligands from ChEMBL, nearly all of which were synthesized years after those used for model construction. Extrapolation across diverse chemical structures was possible because the method addresses the ligand pose problem and provides structural and geometric means to quantitatively identify ligands within a model’s applicability domain. Predictions for such ligands for the four tested targets were highly statistically significant based on rank correlation. Those molecules predicted to be highly active ($\hbox {pK}_i \ge 7.5$) had a mean experimental $\hbox {pK}_i$ of 7.5, with potent and structurally novel ligands being identified by QMOD for each target.

## Introduction

We introduced the Surflex-QMOD method for 3D-QSAR (“QMOD” hereafter) [1] as a more physically meaningful approach than the antecedent Compass approach [2, 3], which itself was offered as a means to improve the fidelity of predictive models to what is understood about protein–ligand binding interactions. We have previously shown that the QMOD procedure is capable of making accurate predictions across varying chemical scaffolds [1], learning non-additive structure-activity relationships [4, 5], guiding lead optimization toward potent and diverse ligands [6], and incorporating information derived from biophysical experiments [7]. The QMOD procedure is complex, combining aspects of molecular similarity, multiple-instance machine-learning, and docking. This complexity has heretofore inhibited widespread application of the approach by large numbers of independent investigators.

Here, we report algorithmic and workflow enhancements that provide a simple procedure for model induction, broad and automatic model application, and interpretation of model predictions. Results are presented on a set of eight biological targets, originally assembled by Sutherland et al. [8]. Seven targets were enzymes, including angiotensin converting enzyme (ACE), acetylcholinesterase (ACHE), cyclooxygenase-2 (COX2), dihydrofolate reductase (DHFR), glycogen phosphorylase B (GPB), thermolysin, and thrombin, and one was a ligand-gated ion channel, the GABA$_{A}$R benzodiazepine site (BZR). Direct comparisons were made to four other QSAR approaches (CoMFA, two versions of CoMSIA, and CMF, reported by Zhokhova and Baskin [9]). In addition, for four targets with ample ChEMBL data, we report QMOD results on diverse ligands that are beyond the reach of many QSAR methods.

The QMOD methodology builds and tests a virtual binding site (a “pocketmol”) in the following six steps:

1. *Initial alignment hypothesis*: Two or three ligands are chosen to serve as a seed alignment hypothesis, derived by maximizing their mutual 3D molecular similarity. This process may be augmented using molecular docking, with similarity being used to identify a clique of suitable mutually-similar poses from the docked collection.
2. *Training ligand alignment generation*: For each training molecule, the initial alignment hypothesis is used to guide the generation of multiple poses (typically 100–200), again using 3D molecular similarity.
3. *Probe generation*: The collection of alignments for training molecules is used to guide the placement of small molecular probes that represent possible constituents of the cognate binding pocket. This set of probes may be filtered using experimental information about the configurations of the binding pocket.
4. *Probe subset selection*: A probe subset forming an initial pocketmol is chosen to optimize multiple constraints: the scores of training ligands against the pocketmol should be close to their experimental values, the mutual similarity of the optimal poses should be high, the spatial redundancy of the probes low, and the total clash between the probes and the poses should also be low.
5. *Iterative model refinement*: The pocketmol is refined by iteration of adjustment of the fine positions of the pocketmol probes (to minimize the deviation of computed training ligand scores to experimental data) and refinement of training ligand poses to identify the optimal fit for each.
6. *Prediction on new molecules*: The final pocketmol serves as the target of a docking-like procedure. New molecules are flexibly fit into the pocketmol, seeking the optimal score subject to constraints on ligand energetics. The result produces a set of poses, each with a score and estimates of prediction quality.

**Fig. 1:**
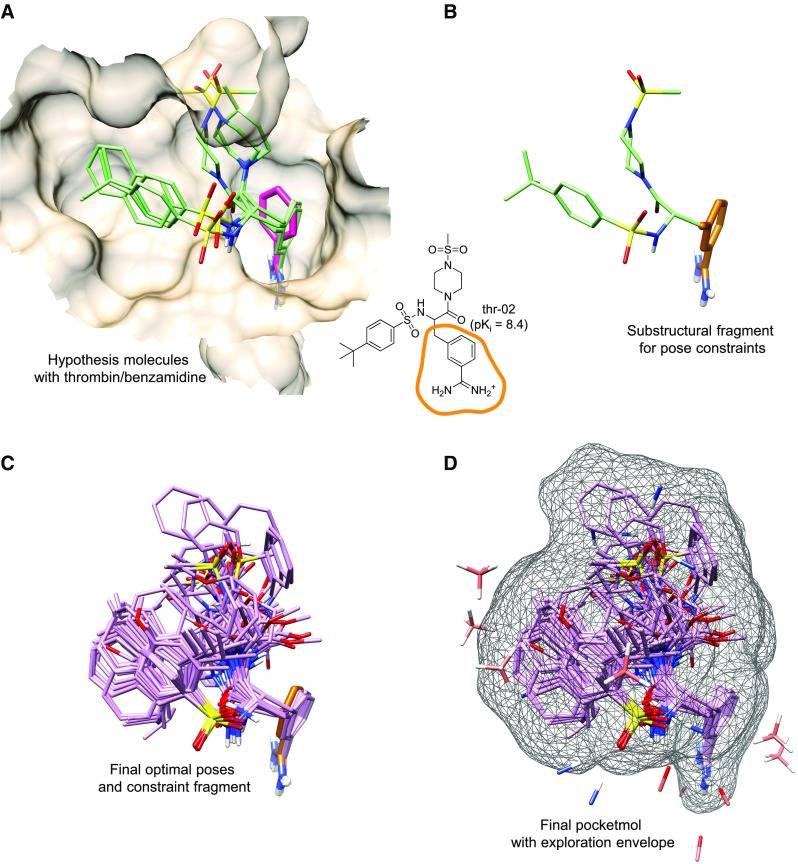
Thrombin model construction: **a** PDB structure 1DWB (thrombin/benzamidine in tan/magenta) shown with the structurally-guided QMOD alignment hypothesis (*green carbons*); **b** the particular pose of thr-02, with its methylene-benzamidine (MBZ) fragment shown with *orange carbons*; **c** final predicted optimal poses of thrombin training ligands (*lavendar*) with the constraining effect of the MBZ fragment; **d** final pocketmol probes (*pink*), optimal poses, and the surface envelope defined by the training ligands (*mesh*)

**Fig. 2:**
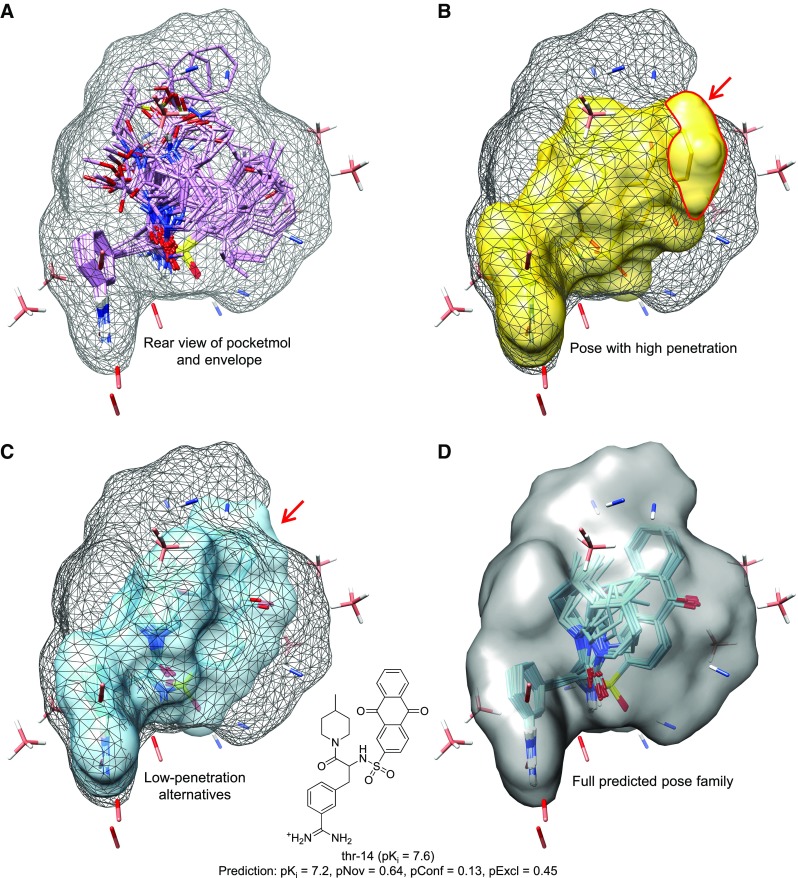
Thrombin model application: **a** rear view of model from Fig. 1; **b** top-scoring single pose of thr-14 (*yellow*) protruding from the exploration envelope; **c** alternative poses compatible with the envelope (*cyan*); **d** full top-ranked pose family shown within the training envelope surface

Here, we report four enhancements (in addition to workflow improvements that are discussed in “Methods, data, and computational protocols”). First, the QMOD procedure is now fully deterministic, with a slow, stochastic, genetic algorithm being replaced by a faster, greedy optimization approach for probe subset selection (Step 4 above). Second, the explored spatial volume from model induction is used to help guide scoring of new ligands. Third, rather than predicting single poses based only on their respective scores, pose *families* are ranked based on probabilistic criteria that combine pocketmol scores with prediction quality metrics. Fourth, greater control is possible over ligand conformational and alignment preferences so that domain knowledge can be used to influence model construction. These enhancements are illustrated in Figs. 1 and 2 using thrombin as a target.

Figure 1a shows three training ligands (the QMOD alignment hypothesis) in the thrombin binding pocket bound to benzamidine. This initial alignment was derived through an ensemble docking procedure [10] that produced 100 poses per ligand, followed by a similarity-driven choice of a single pose for each [7]. The position of the benzamidine fragment of the large inhibitors was shifted from its preferred position when unsubstituted, illustrating the interdependence of substituent changes and molecular alignment. The fine positions of the common substructure shared among the three potent thrombin inhibitors varied as well, though on a smaller scale. The training and testing ligand sets for this target [9] all contained the substructural fragment highlighted in orange in Fig. 1b. For congeneric series, especially flexible ones as in this case, it can be desirable to impose a constraint on the conformation and alignment of a common scaffold.

Here, the particular positions of the methylene-benzamidine from thrombin molecule 2 were used to constrain matching subfragments in training ligands and for new ligands. Figure 1c shows the final optimal poses for all 59 training ligands, which exhibited some minor shifts from the constraining fragment (the penalty for variation is 1.0 kcal/mol/Å$^2$ by default). Figure 1d shows the final derived pocketmol along with the surface envelope of the union of the set of optimal training poses. The C=O probes interacting with the amidine of the inhibitors correspond quite closely to those of the pair of carboxylate oxygens of Asp-189 and the main-chain carbonyl of Gly-219 (detailed protein atoms not shown). The collection of hydrophobic probes enclosing the upper-left of the pocket approximated the shape of the hydrophobic enclosure observed in the actual binding pocket.

The construct shown in Fig. 1d represents the QMOD procedure’s complete model. The model comprises a solution to a multi-factorial optimization problem. The central property is that each training ligand’s maximal pocket interaction score over the collection of poses for that ligand is close to the experimental value. Further, the solution is parsimonious in a quantitative sense: training ligand pairs with similar activity levels will tend to exhibit similar surface shape and polarity. Here, the ligands are quite flexible, and many diverse arrangements of the substituents of the core scaffold are possible. The congruence observed for the sulfonamide linkers and their hydrophobic substituents is a property of the shape and composition of the induced pocket.

Figure 2a shows the reverse view of what is shown in Fig. 1d. To predict binding affinities and poses for new ligands, the model is used in an analogous fashion to a protein structure with a collection of binding poses for ligands whose bound configurations are known. The explored envelope (shown in mesh) is used as an additional soft constraint when fitting new ligands into the pocketmol: penetrations beyond the envelope incur a penalty to encourage identification of solutions that fit within explored space. Figure 2b shows the highest scoring pose for thrombin molecule 14 (predicted $\hbox {pK}_i$ = 7.3), which exhibited a protrusion from the explored training pose envelope (red arrow). Figure 2c shows well-contained poses for molecule 14, the best of which scored slightly lower ($\hbox {pK}_i$ = 7.2).

We have recently shown that binding pose prediction from ensemble docking can be very significantly improved by considering molecular similarity to the bound configurations of prior known ligands [10] (subsequently a related approach was reported by Kelly et al. [11]). We have adapted our approach for docking to the QMOD method in order to improve the reliability of pose and affinity predictions. The details of the computation are presented below, but the principle is that a calculated numerical property of a predicted pose is transformed into a probability using statistics derived from training ligands. Probabilities from three quality metrics are used to adjust the energetic score for each pose. Families of related poses then receive a Boltzmann-derived probability score, and the family with the highest probability is reported. Figure 2d shows the highest-probability pose family for thrombin molecule 14. The predicted $\hbox {pK}_i$ was close to the experimental value (just 0.4 log units low). The three probability-normalized metrics are described in detail below. The first is an estimate of overall molecular novelty that considers the extent to which the full set of training ligands has explored the spatial and compositional characteristics of the new ligand. The second is an estimate of confidence based on the maximal similarity of the new ligand to a *particular* training ligand. The third is a normalized value reflecting penetration into excluded volume beyond the explored training envelope. Molecule 14, judged based on its top-ranked pose family, was not particularly novel when considered in the context of all of the training ligands, nor did it make excessive penetration beyond the training ligand envelope. However, it was not structurally similar to any particular single training ligand, but rather its surface resembled parts of multiple ligands cobbled together, allowing QMOD to produce a good prediction.

Results on the original Sutherland data set were robust, and application of the derived QMOD models to diverse ChEMBL molecules demonstrated practically useful predictive extrapolation. Together with algorithmic and workflow enhancements, these results suggest that Surflex-QMOD will have broad applicability for lead optimization, and the method is being made widely available to independent research groups.

## Methods, data, and computational protocols

Surflex computational methods have been described in detail in previous work: 3D similarity [12, 13], 2D-similarity and computations involving comparisons of single molecules to *sets* of molecules [14, 15], docking (including used of multiple protein structures) [10, 16–18], and both standard and structure-guided QMOD [1, 4, 6, 7]. Details of algorithmic enhancements made to the QMOD procedure will be described in what follows, but prior descriptions will not be repeated except in abbreviated form where needed.

### Molecular data sets

The results in this work were derived from the data summarized in Table 1. Ligand sets for eight biological targets originally assembled by Sutherland et al. (reported in 2004 [8]) were taken from the archive of Zhokhova and Baskin [9]. The overall benchmark consisted of eight data sets that had been curated in order to analyze performance of CoMFA, CoMSIA, and other QSAR methods using *designed* test sets [8]. The targets, target types, and numbers of compounds are listed in Table 1, hereafter referred to as the “Sutherland benchmark.”

The sets were used exactly as structured in the original report in order to facilitate direct comparisons with previously reported results. As described in Sutherland et al. [8], the train/test splitting procedure was designed so as to maximize the diversity of the test set and to examine the predictive accuracy of methods when extrapolating outside the training set. Approximately one-third of molecules for each target were selected by optimization using a maximum dissimilarity algorithm and assigned to the test set, with the remaining compounds assigned to the training set. Selection was optimized under a restraint such that the selected test compounds had a distribution of activities similar to that of the complete set. The structural novelty of thrombin molecule 14 from Fig. 2 compared with typical training ligands (see Fig. 1) is an example of the effect of this procedure. This procedure yields more challenging conditions for predictions than more typical random selection approaches and was intended to measure extrapolative power of QSAR models in order to better reflect future application rather than interpolation. In 2013, the Continuous Molecular Fields (CMF) approach was described in detail using the same data sets with the same molecular poses [9].

**Table 1:** Datasets from Sutherland et al. [8] as used by Baskin and Zhokhova [9]

Target	Target type	Train	Test	Activity range	ChEMBL ID	N ligands
Acetylcholinesterase (ACHE) [19]	Carboxylesterase	74	37	4.3–9.5 (pIC_50_)	220	4910
$\hbox {GABA}_{A}$ receptor (BZR) [20]	Chloride channel	98	49	5.5–8.9 (pIC_50_)	1,907,607	2269
Cyclooxygenase-2 (COX2) [21]	Oxidoreductase	188	94	4.0–9.0 (pIC_50_)	230	5670
Thrombin (THR) [22]	Serine protease	59	29	4.4–8.5 (pK_*i*_)	204	4546
Angiotensin-conv. enzyme (ACE) [23]	Metalloprotease	76	38	2.1–9.9 (pIC_50_)	1808	711
Dihydrofolate reductase (DHFR) [24]	Oxidoreductase	237	124	3.3–9.8 (pIC_50_)	202	974
Glycogen phosphorylase B (GPB) [25]	Glycosyltransferase	44	22	1.3–6.8 (pK_*i*_)	4696	673
Thermolysin (THER) [26]	Metalloprotease	51	25	0.5–10.2 (pK_*i*_)	3392	104

For this work, new test ligand sets and associated activity data for the eight targets were assembled by searching the ChEMBL database for matching targets and then obtaining the sets of compounds with target-associated bioactivities. Table 1 lists the ChEMBL target ID and corresponding number of total assay values (N) for each target. The 4 targets for which there were $>$2000 ChEMBL assays values (ACHE, BZR, COX2, and THR) were used to test the screening utility of our QMOD models (the remaining four each had less than 1000 assay values). The sets of total assay values were filtered to retain compounds with molecular weight between 100–800, and $\hbox {pIC}_{50}$ or $\hbox {pK}_i$ assay values $>$4.0.

Redundancy was eliminated by using an average activity for compounds with more than one assay value. Compounds were considered outliers and therefore eliminated if the difference between the maximum and minimum activity values was greater than 2 log units. A 2D similarity method was used to eliminate compounds identical to those in the training sets. This resulted in ChEMBL datasets for ACHE, BZR, COX2, and thrombin containing totals of 2454, 1158, 2322, and 3097 compounds, respectively.

The existence of multiple assay values for some compounds provides some idea of the expected lower bound on absolute prediction errors for the ChEMBL compounds. The average deviations between minimum and maximum $\hbox {pIC}_{50}$ or $\hbox {pK}_i$ values for the targets ranged from roughly 0.5–1.0 log units *after* eliminating outliers. This is significantly higher that what is seen with biochemical assays conducted within a single laboratory (typically 0.3–0.5 log units).

### QMOD enhancements

There were four major changes made to the QMOD algorithm for the work reported here, detailed as follows.

#### Probe set selection

Given initial ligand alignments (typically 100–200 poses per training molecule) and a large set of possible probes (often many thousand), a probe subset forming an initial pocketmol must be chosen. The initial approach for QMOD to identify a probe subset that approximately satisfied the desired relationship between computed and experimental activities was done using a mixed integer programming solver [1]. That approach required consideration of just *single* poses for each molecule for the initial selection. Subsequently, approaches were implemented that allowed for consideration of full ligand pose pools, where the probe set selection method simultaneously optimized fit to experimental data as well as optimizing multiple constraints, as follows:

1. The scores of training ligands against the pocketmol should be close to their experimental values. Given a particular probe set, the score of each pose of each ligand is computed, and the maximal value is defined as the ligand’s score, with the corresponding pose being considered optimal. Scores can be constrained to be equal to some value (plus or minus a user-settable deviation), or be constrained to be less than or greater than a particular value. The average of the sum of squared deviations beyond desired values (the mean-squared-deviation or *D*) is optimized toward a minimal value.
2. The mutual similarity of the optimal poses of molecule pairs whose activities are close should be high. Similarities of all non-self pairs of optimal poses are computed, with the resulting values being weighted by a Gaussian term (identical activities are weighted 1.0, and differences in activity reduce the weight), and the weighted values are summed and normalized to a maximum value of 1.0. This is the *parsimony* (*P*) resulting from the current probe set configuration [4].
3. The spatial redundancy of the probes should be low. Values are set on the preferred minimal RMS deviation between like-kind probes, below which a positive-valued penalty is incurred. Values may be selected, for example, to skew toward hydrophobic pocket solutions (by limited spatial closeness of polar probes) or toward more hydrophilic (by allowing relatively close positioning of like polar probes). This redundancy value (*R*) is optimized toward a minimal value.
4. The average clash between the probe set and the optimal poses should also be low. This creates a preference for models in which explanations of ligand activity are derived through favorable interactions with pocketmol probes rather than by constructing stiff enclosures. Average clash (a value $C \le 0.0$) is minimized in absolute magnitude.
5. There should be as few probes as possible (*N*) while still meeting the foregoing constraints.

The most sophisticated of these past approaches made use of a genetic algorithm, but this part of the overall QMOD computation was a bottleneck in terms of speed, and the results, being stochastic, were more variable with respect to initial conditions and parameters than was desirable. For this work, the stochastic probe selection method was replaced with a deterministic method, which improves speed and reproducibility. The function that is minimized by compositional selection of probes is as follows:

1 $$\begin{aligned} f = \alpha D + \beta (1-P) + \gamma R + \delta C + \epsilon N \end{aligned}$$

By default, the respective weights in Eq. 1 are: $\alpha =1.0$, $\beta =20.0$, $\gamma =20.0$, $\delta =-1.0$, and $\epsilon =0.03$. A improvement in the overall objective function of 1.0 can be achieved by any of the following: reduction in the deviation of each ligand’s computed activity from experimental by 1.0 $\hbox {pK}_i$ units, an increase in parsimony of 0.05, elimination of a redundant probe whose RMSD from its like-kind neighbor was 0.05 less than preferred, a reduction in average magnitude of clashing of 1.0 $\hbox {pK}_i$ units, or a large reduction in the total number of probes. The high weight $\gamma =20.0$ on probe redundancy amounts to a rule to avoid excessively close probes, while the constraint on the total number of probes is quite weak and simply ensures that a new probe will improve overall fit by at least 0.03 units. Effective optimization of probe set selection in a typical case results in an overall score of $f \approx 6.5$, with the most important constituent terms being the MSE ($D \approx 1.5$), parsimony ($P \approx 0.75$), and average clash ($C \approx -0.1$).

Note that these weights were not chosen systematically using multiple targets. Rather, using the 5HT1a ligand set from the initial QMOD report [1], exploration of parameter values was made with $\alpha$ assumed to be 1.0, and baseline convergence was established with all other values being zero. The other values were then increased in magnitude sequentially (first $\gamma$, then $\beta$, $\delta$, and $\epsilon$) to yield probe selections with comparable convergence in terms of MSE, but where the effects of each additional constraint were maximized. Systematic optimization of these parameters using multiple data sets has not been undertaken and is likely to yield performance improvements.

Carrying out optimization of the objective function is done using a series of greedy procedures:

1. Given the set of initial ligand alignments, find a parsimonious pose clique. This is done by fixing the choice of a single molecule’s pose, then selecting the first pose of each remaining molecule as an initial state. Then, for each pair of molecules, we try all possible pairs of new replacement poses for those two. If the best among the replacement choices is better than the current best, we replace the current solution with using the replacement choices. The process iterates until no replacement results in an improvement in computed parsimony.
2. Given a locally optimal pose clique, identify a *weighted* probe set that optimizes the objective function *f*. Initialize all probe weights to 0. Using a fixed value to change probe weights, systematically alter the weight of each probe by the fixed value, ensuring that probe weights remain on the interval [0, 1]. For each weight alteration, compute *f* and keep track of the best alteration. Make the single best weight change uncovered. Repeat the process for up to 10,000 cycles or until no improvement is possible. The fixed value for probe weight changes begins with 0.2 for one iteration of the procedure, then it is set to 0.1 for one more iteration. The resulting weighted probe vector is locally optimal under changes of 0.1 weight units for any probe.
3. Given the weighted probe solution, open choices for all ligand poses so that now the optimal pose will result from that with maximal score for each molecule. The same procedure just used to optimize probe weights is repeated, but pose choice is now free. In order to limit the computational complexity of this step, only those probes that were “winners” occasionally (“good probes”) in the previous step are considered for weight variation in this step.
4. The real-valued probe weight vector is binarized, and the optimization process above is repeated, but with weight values of only 1 and 0. The final probe set (those probes with weight 1) is locally optimal with respect to the function *f* under any single binary weight change among the good probe set.

As implemented, ten different parsimonious pose cliques are used, each identified by fixing a single pose of either the first or second training molecule to one of the first five poses from the initial alignment process. For each such initial clique, the remaining optimization process is carried out. The result with the lowest overall value of *f* is the solution that is carried forward for further refinement. Additional refinement *does not* vary the composition of the pocketmol, just the fine positions of the probes that have been selected in this step. As the pocketmol is refined, so too are the poses of the ligands.

In cases where selection of an optimal probe set begins from a pre-existing probe set, the procedure is modified slightly, never making use of fixed pose cliques. First, the existing set of probes and poses (whose positions may have changed from the initial QMOD procedure steps) are used in order to find a locally optimal weighted probe vector. Then, all probes are considered in further optimizing *f*, first using real-valued weights and then using binary weights, as above. In practice, large parts of the beginning probe set are retained, having been the subject of previous optimization. The typical composition of the resulting probe set is generally at least 80 % original probes, with a total number of probes slightly larger than the original set.

This is necessarily a complex algorithm, requiring both time and a large memory footprint in order to avoid repetitive computations (one can cache computations of interactions scores between probes and poses as well as similarity scores among poses of different ligands). Further improvements are certainly possible.

#### Exploration envelope

For small, relatively rigid, molecules, the process of alignment and conformational optimization to fit into a pocketmol will not typically identify poses that reach outside of what is seen among a set of similarly-sized training ligands. However, as seen in Fig. 2, as molecules become larger and more flexible, especially when branching creates opportunities for internal clashing, parts of a ligand may protrude into unexplored parts of space where no explicit constraints could have been learned. In order to encourage exploration of poses within the explored training envelope, a small penalty has been introduced during pose optimization. Any atomic extrusion $d_i$ beyond the training ligand envelope incurs a sigmoidal penalty, as follows, for atom *i*:

2 $$\begin{aligned} e_i = \eta \left( 1 - \frac{1}{1 + e^{(d_i+\kappa )/\lambda }}\right) \end{aligned}$$

The constants have not been carefully optimized, and the values used are: $\eta = -2.0$, $\kappa = -0.75$, and $\lambda = 0.15$. With these values, excursions of 0.2 Å receive a penalty of −0.05 and increase to close to −2.0 as they reach 1.5 Å (with the inflection point of the sigmoid being at 0.75 Å). Because any single protrusion is capped at a relatively modest penalty, the structure of this term provides generally non-distortive pressure for ligands to lie within the envelope. However, when a molecule’s score can be significantly improved by including an excursion, such a pose will be retained. As seen in Fig. 2, the presence of a protrusion does not *eliminate* a pose from consideration, but the effect of the envelope surface serves to enhance the exploration of conforming ligand configurations.

#### Pose families and prediction quality metrics

Previous versions of QMOD produced a set of poses for a molecule, ranked only by score against the pocketmol. We have previously used *pose families* consisting of closely-related ligand configurations to represent the results of docking [17]. Recently, we generalized that notion to take advantage of information derived from the known bound configurations of previously studied ligands [10].

Rather than treating the final predicted pool of *n* poses as individual and independent predictions, pose families are constructed based on RMSD, and they are ranked based on Boltzmann-derived probability scores. A given score of *x* for a particular pose family means that it is expected for the experimentally observed bound configurations of the ligand in question to fall within that family with probability *x*. To use information from other ligands, the procedure takes an idea from statistical potentials, which derive energy functions from observed distributions of molecular configurational properties (typically distances). In the case of amino-acid residues, the free-energy of interactions between residue types *i* and *j* is given as follows [27]:

3 $$\begin{aligned} w_{ij} = -R T \ln \left( \frac{\rho _{ij}(r)}{\rho ^*} \right) \end{aligned}$$

The notion is that configurations that are common in the observed data relative to the reference state lead to a high relative likelihood and consequently a favorable negative energy. For docking, we used this idea to provide an energetic correction to predicted molecular poses, where those that appeared to be more “native-like” were treated like favorable amino-acid distances. So, a group of ligand poses that were quantitatively more similar in terms of 3D surface properties to prior ligands would see improvements in their corrected energies, which would then lead to a higher probability for the pose family. Use of this approach led to significant improvements in predictions of bound configurations of novel ligands [10].

Here, we extend this notion to exploit *quality metrics* computed for each predicted pose of a new ligand. Conceptually, more native-like in the context of a machine learning prediction means *closer to* the properties of the training ligands. A direct and obvious metric is the maximal molecular similarity of a test molecule pose to any of the final optimal training molecule poses (we have previously used this as a measure of confidence within QMOD). Suppose we have a collection of predicted pocketmol poses for a ligand, denoted $L_{1\ldots n}$, some of which are more like those seen with training ligands and some not, and this is reflected in the set of corresponding maximal similarities $S_{1\ldots n}$.

We can use a similar formulation to Eq. 3 by expressing the similarities of these poses to training ligand poses in terms of probabilities. In order to do this, we estimate the properties of the similarity distribution among the pairs of optimal final training poses. From prior work, we have found that such distributions tend be normally distributed when using the Surflex-Sim metric, so we estimate $\mu$ and $\sigma$ for the population of all non-self pairs of poses from the end of the QMOD model induction process (the lavendar poses from Fig. 1). So, given a pose $L_i$ for a new molecule, with associated maximal similarity $S_i$, we define a correction to the energy score ($w_i$) as follows:

4 $$\begin{aligned} p_i&= 1 - \frac{1}{2}\left( 1 + erf\left( \frac{S_i-\mu }{\sigma \sqrt{2}} \right) \right) \end{aligned}$$

5 $$\begin{aligned} w_i&= -R T \ln \left( \frac{1}{p_i} \right) \end{aligned}$$

Equation 4 is simply the area under the right-hand side (high similarity) of the observed distribution of similarity values among the training poses. A predicted ligand pose that looks much less like the training ligands than other predicted poses would receive a low similarity score, resulting in a value close to 1 from Eq. 4 and an energetic correction of close to zero. Conversely, a predicted ligand pose that looks very native-like compared with other poses would receive a low probability and a large, favorable energy correction from Eq. 5.

The corrections to pose scores may come from multiple measures, and QMOD currently produces three quality metrics. The similarity measurement just described becomes the probabilistically normalized confidence (pConf) value. The value represents the degree to which a particular new ligand looks quantitatively similar to a *particular* training ligand. A related measurement, called novelty (denoted pNov in its normalized form), represents the degree to which a new ligand looks like what is covered by the *union* of all training ligands. The last measure quantifies the degree to which a particular ligand pose penetrates beyond the training ligand envelope into preferentially excluded space (pExcl). Each of these measures for each predicted pose is compared with information derived from what was observed among the training ligands in order to arrive at probabilistically normalized values, which then are used to adjust pose family probability estimates.

As just described, only the optimal final pose of each training ligand is used to estimate the distributions for each quality metric. Because the QMOD learning procedure maintains a pose pool for each training ligand, it is possible to obtain more robust estimates for the distributions by considering all poses for each training ligand that either geometrically close (by RMSD) to the optimal pose or are close based on pocketmol score (the RMSD threshold is 1.0 Å and the score deviation threshold is 2.0 $\hbox {pK}_i$ units). For large training sets, this makes little difference, but for smaller training sets, the effects of single molecules that may behave as outliers is minimized.

For a new ligand in the scoring process, multiple pose families may be reported. For each, the ligand score that is reported corresponds to the maximal (unadjusted) score for any pose within the family. The reported pConf, pNov, and pExcl values are the mean values from all poses within the family. High confidence, low novelty, and minimal penetration into excluded volume all tend to correlate with lower prediction errors. In practice, thresholds of pConf > 0.35 (high confidence), pNov < 0.85 (low novelty), and pExcl < 0.95 (non-extreme exclusion penetration) are used to identify subsets of ligands on which predictions may be considered to be more accurate.

Recall from Fig. 2 that the single top-scoring pose was *not* part of the top-ranked pose family, due to placement of the sulfonamide substituent in a manner that deviated from what had been observed in training. It is important, however, to note that use of this re-ranking approach is necessarily heuristic. It may be the case that a pose for a new ligand that is discordant with respect to similarity or exclusion envelope penetration is, in fact, closer to physically correct than a concordant one. However, it is likely that, in most cases, on ligands for which a reasonable prediction might be expected, that the best prediction will derive from a set of poses clearly similar to those observed from training and which fall within the training envelope.

#### Constraints on conformation and alignment

A user may specify a constraint on either the conformation of a substructure or may constrain both the conformation and alignment of a substructure. The former is useful in cases where detailed knowledge of the energetics of a particular system provide a more accurate geometry than the QMOD internal forcefield. The latter is useful when either there is specific knowledge of the binding preference of a particular moiety or where learning convergence is otherwise difficult to obtain. Cases where flexible molecules all share a common core element that does not vary can lead to underconstraint in model-building.

In the case of thrombin, illustrated in Figs. 1 and 2, model convergence was poor using a purely unconstrained pocket induction procedure. However, the favored position of the common benzamidine fragment was useful as an “anchor” which led to adequate convergence. The constraints can be upon multiple different substructures, and penalties from deviation are specified in terms of $\hbox {pK}_i$/Å$^2$ (default is −1.0 for both conformation and alignment constraints). As seen in the thrombin example, movement of the constrained fragment does occur in a context-dependent manner for different ligands. It need not be the case that either all of the training or all of the new molecules contain fragments to be constrained.

### Protein structure guided hypotheses

For all targets but BZR, protein structural information was available, and it was used for the generation of initial alignment hypotheses (Step 1 from the Introduction). Protein structural information was not used in any other fashion to influence the resulting models. Protein structures were downloaded from the Protein Data Bank as biological assemblies. Ensemble docking for generation of the seven structure-guided QMOD hypotheses employed five structures for each target: (1) ACE structures 1UZE, 1UZF, 2C6N, 2OC2, 3L3N, (2) ACHE structures 1MAA, 2GYU, 1Q83, 1Q84, 2GYW, (3) COX2 structures 1PXX, 3LN1, 3NT1, 3RR3, 4COX, (4) DHFR structures 1DRF, 2DHF, 1HFR, 1KMS, 1MVS, (v) GPB structures 2F3P, 1AXR, 2GPA, 1XL0, 1NOI, (vi) thermolysin structures 1QF0, 4TMN, 2TMN, 1THL, 1HYT, and (vii) thrombin structures 1K21, 1CA8, 1DWB, 1BMN, and 1D3P.

The results of ensemble dockings were used as input to an automatic procedure that selects poses maximally similar to one-another and also to other native ligands. Procedures for docking [10] and for identifying an alignment hypothesis based on the combination of docking and molecular similarity [7] have been described in detail previously.

### Computational procedures

The QMOD results reported here were generated using Surflex-QMOD version 2.039, which includes all of the algorithmic enhancements described. The current release (v3.065) makes improvements in workflow, moving from a script-based approach to a small number of simple commands. The current release exactly reproduces scoring of new molecules with the resulting models, and these are available in the data archive associated with this paper. Model induction results are statistically equivalent between the versions, but they differ slightly due to changes in default parameters. Details of the precise computational procedures are available in the data archive.

Briefly, the procedure for producing a QMOD model is as follows (illustrated using the v3.065 version):

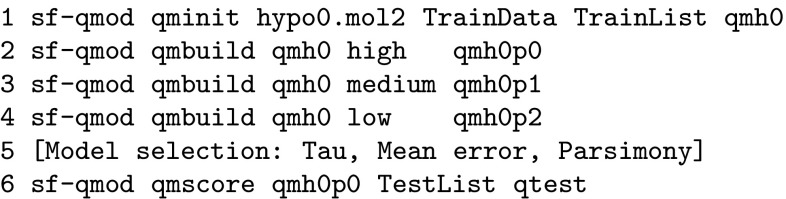



Line 1 builds the initial ligand alignments and produces the overall probe set (Steps 2 and 3 from the Introduction) using an alignment hypothesis (Step 1). The next three lines build full QMOD pocketmols (Steps 4 and 5) using three different density values for polar probe selection. In the case of thrombin, the option “-qmatch mbz-frag.mol2” was used in order to enforce the pose constraint depicted in Fig. 1. For COX2 and DHFR, each with large training sets (188 and 237 molecules, respectively), initial models were built from a fraction of the training sets, and final models were constructed by iterative incorporation of the remaining fractions, using the “qmadd” procedure (see data archive for details).

For all but COX2 and DHFR, model selection from among the three generated was done using the three training metrics of Kendall’s Tau, average error, and parsimony, with each yielding a “vote.” The winner was selected as the preferred model for testing (no ties were observed). For COX2 and DHFR, each partially trained model was tested on the next fractional training molecule set. Rank-correlation predictive performance of the penultimate trained models on the final fractional training set was used for model selection.

For the benchmark data set, results are presented for all test molecules (see Table 1 for counts) using the “qmscore” (Line 5 from above) with the selected model. QMOD pocketmols may be applied to new molecules of widely varying structures, but, depending on the diversity and coverage of the training set, the reliable domain of applicability varies from model to model, and predictions on ligands of some structural classes may be more accurate than others.

For the four targets with ample ChEMBL data, the selected models were tested on data of much more diverse chemical structural variation than represented in the benchmark test data. We use the term “in-model” to describe those molecules for which the QMOD activity scores are most reliable. The production of distributionally normalized novelty, confidence, and exclusion values (pExcl, pConf, and pNov) allows for unbiased selection of molecular subsets. For ACHE, BZR, and COX2, in-model molecules were defined as those predicted by QMOD with pNov < 0.85. However, in the case of thrombin, the new ChEMBL ligands were so different from those seen in training that *none* of the molecules passed this threshold. The in-model definition for thrombin for a new molecule relied on raw final reported values of similarity and exclusion penalty (>0.70 for similarity and >−0.40 for exclusion penalty).

Scaffold novelty was characterized using 2D comparisons of test ligands to the full set of training ligands for a particular target. The calculation has been previously described [14], and it makes use of probabilistically normalized 2D similarity values that are transformed into a single log-odds score using the multinomial distribution. Large, positive values indicate high likelihood that a particular ligand is topologically similar to the set to which it was compared.

Data and computational protocols are freely available by download. Software is available by request. Details may be found at www.jainlab.org.

### Statistical analysis

The primary results of QMOD model performance, both on convergence during training and on quality of test molecule predictions are reported using Kendall’s Tau ($\tau$) [28] and mean absolute error. The former is a non-parametric rank-correlation statistic on the interval $[-1,1]$ whose meaning is intuitive: a value of 1 indicates equivalent ranking between predicted and experimental values, a value of -1 indicates reversed ranking, and a value of 0 indicates no correlation of ranks. For this work, values are considered tied if they differ by 0.1 or less, unless otherwise specified. Statistical significance of $\tau$ can be computed analytically for large sample sizes, but in this work, significance has been assessed used permutation analysis (with 10,000 permutations). The advantages of $\tau$ over the widely used Pearson’s correlation (*r* or $r^2$) include dependence only on ranks, invariance to increasing monotonic transformations, and robustness against outliers [29].

Reports of QSAR performance often make use of a term for test set performance that is *not* Pearson’s correlation, but which is also denoted $r^2$ or $R^2$ (or $q^2$ for the analogous case of model cross-validation using a leave-one-out scheme), popularized by use in the initial report of the widely used CoMFA technique [30]. Here, these values will be denoted $R^2_{pred}$ and $q^2$ to distinguish from Pearson’s $r^2$. Recall that the values are defined as follows, where $x_i$ are experimental activity values and $y_i$ are the predicted values:

6 $$\begin{aligned} R^2_{pred}&= 1 - \frac{\sum \nolimits _{i=1}^{n}(x_i-y_i)^2}{\sum \nolimits _{i=1}^{n}(x_i-\bar{x})^2} \end{aligned}$$

7 $$\begin{aligned} r^2&= \frac{\left( \sum \nolimits _{i=1}^{n}(x_i-\bar{x})(y_i-\bar{y})\right) ^2}{\sum \nolimits _{i=1}^{n}(x_i-\bar{x})^2 \sum \nolimits _{i=1}^{n}(y_i-\bar{y})^2} \end{aligned}$$

Equation 6 normalizes the predicted residual sum of squares (numerator, denoted “PRESS” in the original report of the partial-least-squares method [31]) by the spread in the data to be predicted (the denominator) [30]. The formulation was introduced to characterize performance of PLS under cross-validation. However, in the case that predicted values have an equivalent mean to experimental values and the slope of a line fitted against predicted/experimental values is one, the $R^2_{pred}$ and $r^2$ values are equivalent (otherwise $R^2_{pred}$ becomes smaller than $r^2$). One distinct advantage of the Pearson formulation is that it is invariant to linear transformations, and while being subject to poor behavior with respect to outliers, it has good statistical properties in many situations. It has a direct relationship to linear regression, in that $r^2$ explains the proportion of variance explained by the linear regression of *y* on *x* or vice-versa.

$R^2_{pred}$ can be made *equivalent* to $r^2$ by a linear transformation of each $y_i$ to $\hat{y}_i$ as follows:

8 $$\begin{aligned} b&= \frac{\sum \nolimits _{i=1}^{n}(x_i-\bar{x})(y_i-\bar{y})}{\sum \nolimits _{i=1}^{n}(y_i-\bar{y})^2} \end{aligned}$$

9 $$\begin{aligned} a&= \bar{x} - b \bar{y} \end{aligned}$$

10 $$\begin{aligned} \hat{y_i}&= a + b y_i \end{aligned}$$

Brown and Muchmore [32] made this transformation to predicted binding affinity values from MM-PBSA in order to produce interpretable statistics on prediction deviations (the center and slope of the values resulting from physical simulation calculations were far from experimental values).

**Table 2:** Training results for the complete Sutherland benchmark

	QMOD				Fixed-alignment methods		
	$\tau$	$\tau$ *p* val	Avg Err	$r^2$	$q^2$	$q^2$ CI	SD
ACHE	0.60	<0.001	0.65	0.59	0.52	0.43–0.61	0.05
BZR	0.62	<0.001	0.38	0.52	0.40	0.29–0.50	0.05
COX2	0.51	<0.001	0.65	0.40	0.52	0.38–0.65	0.07
THR	0.66	<0.001	0.48	0.65	0.67	0.54–0.80	0.07
ACE	0.69	<0.001	1.01	0.73	0.68	0.62–0.74	0.03
DHFR	0.56	<0.001	0.88	0.54	0.57	0.41–0.72	0.08
GPB	0.39	<0.001	0.69	0.43	0.54	0.27–0.81	0.13
THER	0.70	<0.001	1.09	0.65	0.54	0.46–0.62	0.04

**Table 3:** Test results for the complete Sutherland benchmark

	QMOD				Fixed-alignment methods		
	$\tau$	$\tau$ *p* val	Avg Err	$r^2$	$R^2_{pred}$	$R^2_{pred}$ CI	SD
ACHE	0.60	<0.001	0.68	0.56	0.50	0.31–0.69	0.10
BZR	0.42	<0.001	0.65	0.27	0.10	−0.07 to 0.27	0.08
COX2	0.39	<0.001	1.01	0.22	0.21	−0.10 to 0.51	0.15
THR	0.51	<0.001	0.69	0.42	0.61	0.53–0.69	0.04
ACE	0.39	<0.001	1.72	0.32	0.54	0.39–0.69	0.08
DHFR	0.55	<0.001	1.04	0.46	0.60	0.45–0.69	0.06
GPB	0.50	0.001	0.67	0.46	0.47	0.39–0.55	0.04
THER	0.42	0.002	1.63	0.39	0.44	0.20–0.67	0.12

Here, rather than making explicit transformations on predicted values, we report $r^2$ values for the QMOD results and $R^2_{pred}$ and $q^2$ results from the original reports of performance for other methods. Because those methods are fundamentally regression-based, and because the statistics of the activity values for test data were carefully controlled, substantial differences between the two assessment types are not likely. We report $r^2$ for QMOD, which provides statistically meaningful values and also produces a sensible comparison to results from the prior work. Numerical differences between $r^2$ and $R^2_{pred}$ were small, and conclusions about methodological comparisons were done using the same metrics. Our hope is to encourage the field to make use of *bona fide* statistics that have tractable interpretations with respect to significance [33].

## Results and discussion

The primary data set for this work (the Sutherland benchmark) was curated as a test for QSAR methods, with an emphasis on diverse targets and challenging blind test ligands [8]. In the original report, ligand conformation and alignment questions were addressed manually for 3D QSAR methods. The procedures employed for the eight targets were both involved and target specific, in some cases making use of information regarding known bound ligand poses (e.g. thrombin) and in some cases making alignments without using knowledge of binding modes (e.g. ACHE). In all cases, the alignments were carefully curated in order to yield consistent substituent-based correspondences.

The goal here was to analyze the performance of the QMOD approach using automatic procedures to derive molecular conformation and alignment, both for the model-building process and for scoring new ligands. For the blind test data from the Sutherland benchmark, direct comparisons were made to the performance of four methods, all of which relied on the same *ad hoc* fixed alignment procedures (CoMFA, two versions of CoMSIA, and CMF) [8, 9]. In addition, the QMOD models were tested more thoroughly on new datasets with molecules both structurally and temporally distant from the training sets, curated from ChEMBL.

There were three primary results of this study. First, the eight targets represented in the benchmark dataset were the most diverse set tested to date for QMOD. Performance for QMOD, using a physically realistic, automated, and model-driven alignment method, was comparable to the methods that relied upon manual ligand alignments. For all eight targets, QMOD produced statistically significant rank correlations for predicted activities of test compounds. Second, the QMOD ligand alignments, in all cases, were significantly different from the manual alignments used by other methods. For the enzymes, where crystallographic data were available, the QMOD alignments were physically plausible in all seven cases, whereas the manual alignments clearly were not for multiple targets. In the remaining case (BZR), the QMOD alignment was very different from the manual diazepine-based one, but the QMOD model corresponded well to a homology-based BZR binding site structure. Third, for the four cases where ChEMBL data were plentiful, QMOD predictions were statistically significant in their correlation with experimental activities, and QMOD was able to identify potent and structurally novel ligands reported years in the future from the ligands used for training.

Tables 2 and 3 show the training and test results for QMOD and the average of the fixed-alignment methods. Summary statistics are given for the four fixed-alignment methods for two reasons. First, the inter-target variation in performance was much larger than the inter-method variation (single factor ANOVA yielded a *p* value >0.5 for both training and testing performance). Second, the focus here is not on direct comparison of particular methods. Rather, the question is whether automatic and generally applicable alignment and conformation selection can provide robust performance on challenging QSAR data sets *and* produce practically useful results on structurally diverse ligands that would not be easily modeled using widely used QSAR approaches.

For the training performance measures, QMOD’s results come from a de novo re-fit of training molecules to the induced model (not from cross-validation). This is a measurement of model convergence, and it is not intended to be a direct estimate of future predictive accuracy. In all cases, QMOD derived models converged. When training molecules were fit into the pocketmol in order to optimize binding interactions through variation of conformation and alignment, the scores resulting from identification of the top-ranked pose families were close to experimental values. In all cases, the ranking produced was highly statistically significant. The fixed-alignment methods all produced similar results with respect to internally optimized cross-validation performance (with a somewhat higher level of variation in the case of GPB).

As shown in Table 3, QMOD produced statistically significant rank correlations for all eight targets on the Sutherland benchmark’s designed test sets. In terms of average error, four cases yielded deviations of less than 0.7 $\hbox {pK}_i$ units (1.0 kcal/mol), two of 1.0 units ($<$1.5 kcal/mol), and two produced significantly higher mean error values (equivalent to 2.2–2.3 kcal/mol). Note that in a case such as BZR, with a limited range of experimental activity values relative to expected assay noise, measures such as Pearson’s correlation may not be as reflective of predictive power as rank correlation measures such as $\tau$. For the thrombin case, the fixed-alignment methods produced high performance with very little inter-method variation, appearing to show a marginal advantage over QMOD. However, taken together, characterized by $R^2_{pred}$ for all of the individual methods (data not shown), the blind test results reflected much greater inter-target variation than inter-method variation ($p > 0.5$ by single-factor ANOVA). In direct head-to-head comparisons, QMOD did not consistently outperform any other method, nor *vice versa*, either when comparing raw $R^2_{pred}$ values (without considering confidence intervals) or when comparing such values in the context of confidence intervals computed by permutation analysis for QMOD (in this latter case, there were very few differences to count).

While there was not a numerical performance advantage for QMOD within this set of blind tests, the fact that the method addresses the conformation and alignment problem in a general and automatic fashion is a distinct advantage. Sutherland et al. [8] noted that, while the field-based 3D QSAR methods performed generally better than 2D or 2.5D methods, the methods are not tractable for screening large collections of compounds (even of congeneric series) due to the “manual labor involved in aligning structures.” In what follows, we describe each of the QMOD models, with particular attention to the relationship between the induced model and what is known about the actual binding sites. The congruence of models with the physical basis for binding interactions between ligands and their target proteins is reflected in the degree to which models were able to quantitatively predict activities for structurally novel compounds from ChEMBL.

In what follows, the four targets for which ample ChEMBL data were available will be discussed first, including examples of extrapolative predictions. The overall summary of performance on the ChEMBL experiments is next, and the remaining four targets are discussed last.

### Acetylcholinesterase (ACHE)

The mammalian acetylcholinesterase (ACHE) pocket contains a narrow gorge about 20 Å deep and is comprised of subsites including the peripheral anionic site at the gorge entrance, an oxyanion hole along one wall of the gorge, and the catalytic triad plus quaternary ammonium group interaction site at the bottom of the gorge [34–36]. Construction of an initial alignment hypothesis from which to induce a QMOD model can be derived by using molecular similarity alone. However, as we have previously shown, structural information from protein–ligand complexes can be utilized and benefits model performance [7]. For all targets except BZR, structural information was used for initial alignment derivation (see “Methods, data, and computational protocols” for details) but was not used in any other manner.

**Fig. 3:**
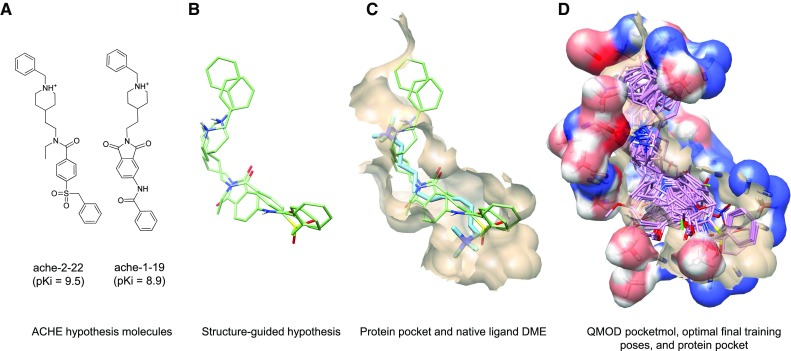
ACHE QMOD model: **a** 2D structures of the training molecules used for the ACHE hypothesis; **b** structure-guided alignment hypothesis (*light green*); **c** alignment shown with the ACHE pocket of 1MAA (*tan*) and native ligand (*cyan*); **d** optimal final poses of the training ligands (*purple*) in the QMOD pocketmol (probes and surface in atom color), with the 1MAA pocket (*tan*)

Figure 3 depicts the overall QMOD model induction for ACHE, with the native protein pocket shown for comparison. Figure 3a shows the structures of the two ACHE ligands used to generate the ACHE alignment hypothesis that is depicted in Fig. 3b (light green). The correspondence between primary features of the ligand pair is sensible, with the amines superimposed, the hydrophobic portions occupying shared volumes, and additional close correspondence between carbonyl oxygen atoms. However, precise atomic correspondence of specific substituents such as the benzyl groups does not occur due to differences in the flexibility of the central linker and overall ligand size. Whereas manual alignment procedures seek to enhance such correspondence, a physical solution to the mutual in-pocket superimposition of the two ligands identifies room for variation. The native ligand of 1MAA (Fig. 3c) follows a similar binding pattern across the middle of the gorge, but it differs in composition and also in how it occupies the ends of the pocket. It has quaternary ammonium ends that contact the indoles of Trp286 and Trp86 connected by an intervening 10-carbon methylene chain [37]. Figure 3d shows the ACHE pocketmol probes and surface surrounding the optimal learned poses of the training ligands. The congruence of the learned pocketmol to the protein pocket (tan surface) is evident.

**Fig. 4:**
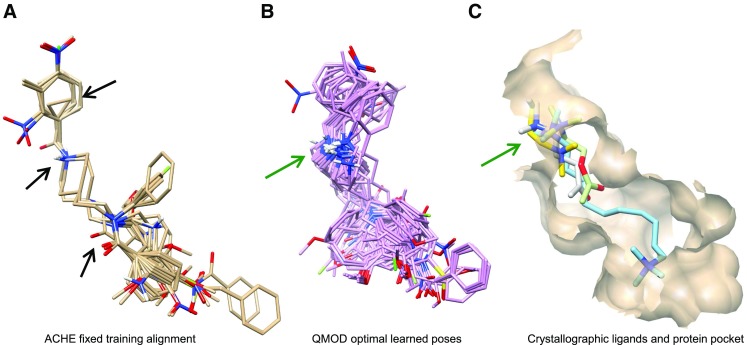
QMOD ACHE training ligand poses and screening utility: **a** Pharmacophoric manual alignment superimposing the benzyl, charged nitrogen, and carbonyl groups; **b** QMOD optimal alignment of training molecules; **c** ACHE 1MAA pocket (*tan*), native ligand DME (*cyan*), and the native ligands of 3 aligned ACHE pockets not used in study (2HA0-CHH in *white*, 2HA4-ACH in *light green*, and 2HA5-ETM in *yellow*)

Both the initial alignment hypothesis and the final optimal ligand poses exhibited the characteristics seen when looking at native binding modes for different ligands of the same pocket. Figure 4 shows the manual fixed-alignments alongside optimal QMOD poses and four native ligands of the ACHE pocket in their experimentally determined poses. The manual alignment procedure employed three pharmacophore features: the benzyl group, the charged nitrogen, and the carbonyl (black arrows), achieving very tight correspondences. This atomic congruence is not reflected in the relative positions of the crystallographic ligands. All four contained quaternary ammonium nitrogens, but none of them bound exactly the same way despite being flexible and not sterically constrained. The QMOD alignments were consistent with the variation observed experimentally among ligand variants in a common pocket.

For ACHE, the initial ChEMBL similarity screen (see “Methods, data, and computational protocols”) yielded a set of 342 ChEMBL molecules. Of these 162 met the criterion for being “in-model” (the novelty measure, pNov, was less than 0.85). For this set, $\tau$ was 0.36 (ties were set at 0.5 $\hbox {pK}_i$ units due to assay variability, $p \ll 0.001$), with mean absolute error of prediction of 1.2 log units. These performance statistics were lower than for the Sutherland test set, but the structural diversity was much higher (an average 2D log-odds similarity to the training set of 66.4 compared with the benchmark test set’s value of 94.0). These included examples with significant structural diversity, especially with respect to the bottom portion of the ACHE ligands from the training set.

**Fig. 5:**
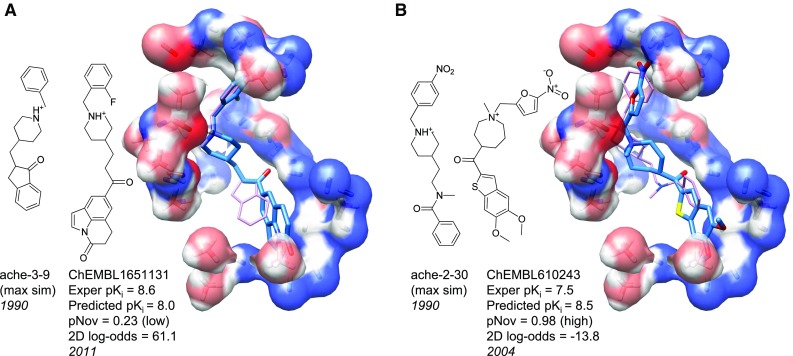
QMOD predictions on structurally novel ACHE ligands from ChEMBL

Figure 5 shows ChEMBL1651131, reported $\sim$20 years after the most similar training molecule. The compound was typical of the structural diversity of the in-model ChEMBL compounds. Of the 28 in-model molecules predicted to have activity $\ge$7.5 (called “winners” hereafter), the mean experimental activity was 7.6. When considering all 342 molecules (not just the in-model ones), prediction quality metrics dropped slightly ($\tau$ of 0.34 and mean error of 1.4), but greater structural diversity was explored. Within this set, 43 molecules were predicted to be winners, and their mean experimental activity was 7.2. When considering this larger set of molecules, QMOD identified much more structurally diverse ligands (see Fig. 5b). ChEMBL610243 exhibited great structural deviation from the training ligands, as reflected in the negative 2D log-odds value.

The degree to which predictions of high activity are believed enough to warrant follow-up, either by synthetic chemistry effort or compound acquisition, is influenced by the evidence supporting the prediction. The QMOD method is not a black-box predictor of activity, but rather constructs a physical model. Because it provides not just a numerical prediction but also an associated set of predicted poses, judgment can be brought to bear on particular compounds, and design ideas can be stimulated. In Fig. 5a, the predicted pose of the ChEMBL ligand follows that of the top half of its near-neighbor training ligand, including correspondence of a common carbonyl/pocket interaction. The space explored by the other training ligands (see Fig. 3) provides a basis to believe that space exists for the large, rigid substituent. Quantification of the nominal novelty of the compound in the context of the full training set increases confidence in the prediction. In Fig. 5b, the predicted ligand has a much higher degree of structural deviation from the set of training ligands, but the quaternary amine corresponds with experimentally observed positions of similar fragments. Further, other interactions (the nitro-group and the carbonyl) recapitulate interactions observed in known ligands. The compound on the left would plausibly make a case for synthesis, and the compound on the right would make a case for experimental testing.

The analysis of ChEMBL results, thus far, has only considered a set of molecules with measurable ACHE activity. A critical question for large-scale application of activity prediction models to guide synthetic chemistry exploration is that of false positive rates. We identified a randomly selected set of 15,515 ZINC drug-like molecules to be used as decoys for assessing false positive rates under the assumption that it is unlikely that such a set would contain a significant proportion of true ACHE ligands with activity $\ge$7.5. Using exactly the same procedures as for the ChEMBL scoring, the fraction of decoys that were both in-model and were scored as nominal winners was 0.013 %. From the full set of ACHE ChEMBL compounds (including those that did not pass the similarity screen), QMOD identified 3.7 % of the true winners, yielding an enrichment rate of nearly 300-fold, which is much better than typically seen for virtual screening using docking approaches. For the subset of compounds that passed the similarity screen, QMOD identified 20.9 % of the true positives.

Identification of less than half of the true positives is not ideal, but from the perspective of real-world use in lead optimization or scaffold replacement, performance at this level would appear to be of practical benefit. The prior marginal value of an untested and unsynthesized compound must be considered to be relatively low, so problems involving false negatives are not as important as problems involving false positives. If a large proportion of relatively inactive ligands were overpredicted, or if even a nominally small fraction of *totally* inactive compounds were predicted as winners, then whatever small number of true positives that exist among a predicted set of winners would be overwhelmed, resulting in a low fraction of successes.

Large-scale application of the ACHE QMOD model considered a total of 2454 ChEMBL molecules with at least some ACHE activity and 15,515 ZINC decoys likely to have no activity (or very poor activity). Of this total set of nearly 18,000 molecules, among the in-model predicted winners, over half had activity $\ge$7.5, whereas less than 3 % of the overall compound set comprised true winners.

### $\hbox {GABA}_{A}$ receptor (BZR)

**Fig. 6:**
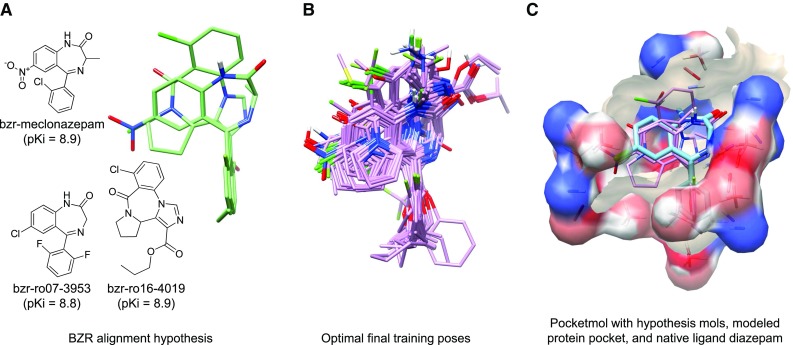
BZR QMOD model: **a** 2D structures of the training hypothesis molecules and the alignment generated by 3D-similarity (*light green*); **b** optimal final poses of the training ligands; **c** pocketmol probes and surface (*atom color*) with the hypothesis molecules (*purple*), and the surface of the homology model of the $\hbox {GABA}_{A}\hbox {R}$ (*tan*) with docked diazepam (*cyan*)

The BZR dataset contained molecules with the classic benzodiazepine scaffold as well as molecules with the seven-membered ring nucleus fused with various heterocyclic rings. For our BZR model, with no experimentally determined protein structures, the standard *de novo* QMOD procedure was used to generate an initial alignment hypothesis, which is based on 3D molecular similarity. As seen in Fig. 6a, three BZR training ligands (meclonazepam, ro07-3953, and ro16-4019) representing both the classic and fused ring scaffolds were used to build the hypothesis (light green). Importantly, the relative poses of the scaffolds was very different from that observed by enforcing a strict atomic congruence of common ring systems, as had been done for the fixed-alignment methods (not shown). The correspondence between the ligands that arose was somewhat surprising. In general, for cases where issues of alignment are not easily resolved, multiple alignment hypotheses may be employed, with the model selection being driven by considerations including model parsimony, convergence, and testing on new ligands.

Another consideration, which we have not quantitatively explored, is “frustration” with an alignment hypothesis during model-building, where final optimal poses change significantly from initial ones. In this case, poses shifted very little. Figure 6b shows the optimal final poses for all 98 BZR training ligands, and the probes and surface of the BZR pocketmol are shown in Fig. 6c. Recently, the X-ray structure of a glutamate-gated chloride channel was used to construct a homology model of this binding site, which was used to dock diazepam [38]. The QMOD BZR pocketmol was superimposed on the homology model pocket, and Fig. 6c shows the final poses of the hypothesis training molecules (purple) as well as the surface of the homology model of the BZR with docked diazepam. Although no protein structure was used in our BZR procedure, there is a striking similarity between the pose of the docked diazepam in the BZR homology model to the poses of the ligands with classic scaffolds in our final training poses.

**Fig. 7:**
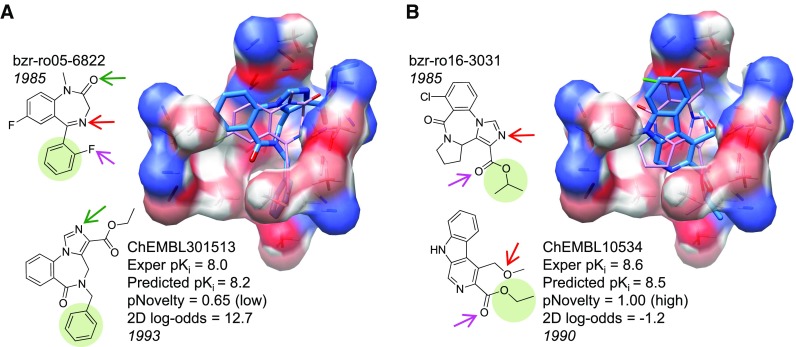
QMOD predictions on structurally novel BZR ligands from ChEMBL

As with ACHE, we followed a similar procedure to screen a set of ChEMBL BZR ligands with known activity and the set of ZINC decoys, the statistics of which will be discussed later. Figure 7 shows two examples of accurate extrapolative predictions using the BZR model. At left is an in-model ligand whose 2D structural similarity to the training ligands was still much lower than seen within the Sutherland test set (a 2D log-odds of 12.7 compared with an average of 40 for the benchmark test molecules). The predicted pose matched the binding mode of flumazenil, a closely related compound, from a recent homology-based study of BZR that employed docking [39]. The carbonyl of the training compound along with the unsubstituted nitrogen of the ChEMBL compound (marked with green arrows) correspond in the QMOD alignment, and both interacted with the hydroxyl of Thr142 in the homology-based prediction. In the QMOD predicted alignment, the phenyl rings (shaded in green) corresponded exactly with one another. The manual alignment rule used in the previous work would not make these correspondences, instead aligning the key carbonyl of the training compound with a carbon within the imidazole of the ChEMBL compound, and producing a geometry where the two phenyl groups cannot be superimposed.

In Fig. 7b, a structurally novel extrapolation is shown, with indications of corresponding parts among the predicted poses of all of the ligands. For this ChEMBL compound, the manual alignment rule from the previous work is simply not applicable. However, the QMOD procedure identifies a pose of ChEMBL10534 that has a rational relationship to the other scaffolds within the model. This $\beta$-carboline compound resulted from an attempt to discover full BZR agonists that were structurally unrelated to the classic benzodiazepine scaffold. [40]. Its negative 2D log-odds score indicates no topological similarity to the training compound set.

The BZR target was, along with COX2, the most challenging for all methods with respect to performance on the test set in the Sutherland benchmark. Here, on the structurally diverse set of ChEMBL compounds, many of which represent *future* predictions, statistical performance was excellent. For the 129 in-model ligands, $\tau$ was 0.55 ($p \ll 0.001$, ties considered at 0.5 log units), with a mean error of 1.0 log units. For the larger and much more diverse set of 843 compounds that included out-of-model structures, $\tau$ was significantly worse (0.17) but was still highly statistically significant ($p \ll 0.001$), with a mean error of 1.4 log units. Consideration of less stringent thresholds on molecular novelty produced performance between these values. Of the in-model ligands predicted to be winners, the mean activity was 7.8, and for the set including out-of-model compounds, mean activity of predicted winners was 7.2

### Cyclooxygenase-2 (COX2)

**Fig. 8:**
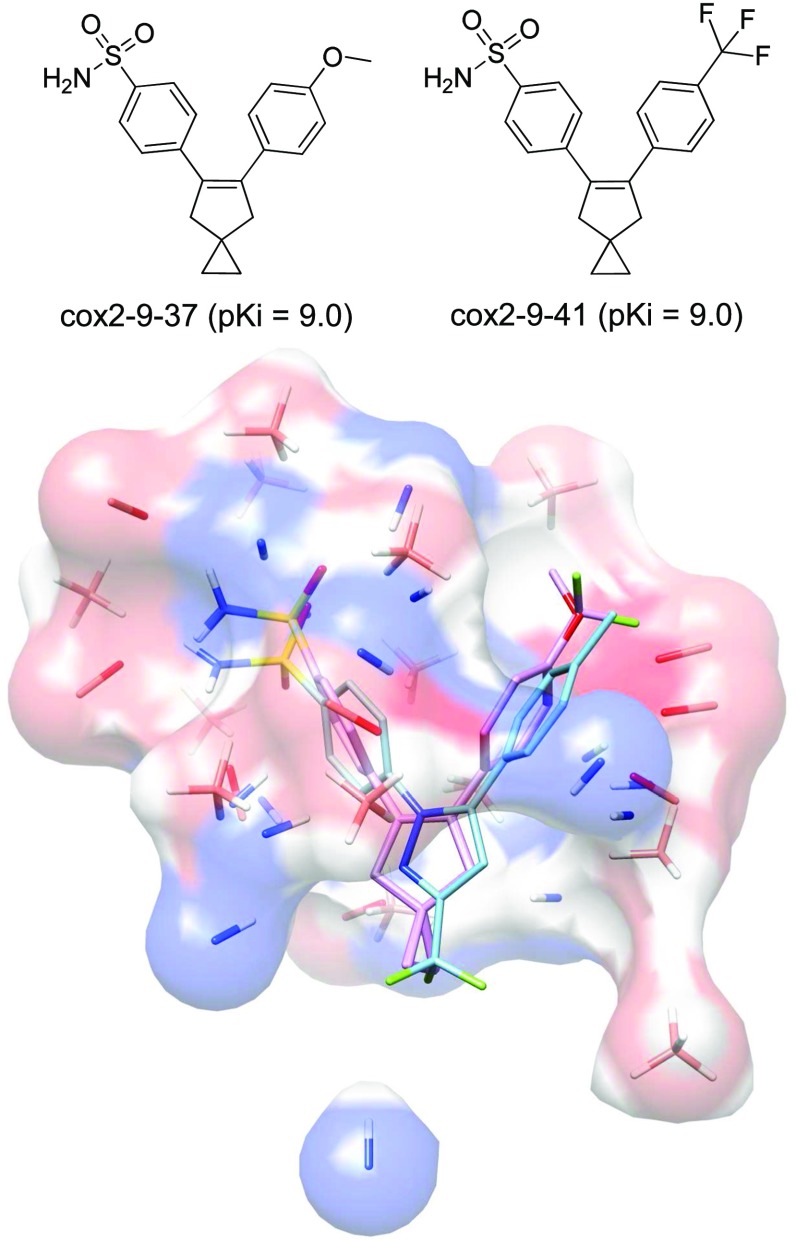
COX2 QMOD model: 2D structures of training molecules for COX2 hypothesis (*top*) and COX2 pocketmol (probes and surface in *atom color*) with the hypothesis molecules (*purple*) and the native ligand celecoxib (*cyan*) from structure 3LN1

The COX2 dataset was comprised of compounds in several structural families grouped according to the central scaffold (e.g. pyrrole, imidazole, cyclopentene, pyrazole, and isoxazole). Nearly all inhibitors had phenyl-sulfonamide substituents (with a few containing phenyl-methyl-sulfone substituents), all were quite rigid, and large variations in activity hinged upon differences in the presence or absence of halogens on the non-sulfonamide substituents of the central ring system. Alignment for COX2 was carried out using the standard structure-guided protocol described above for ACHE. Figure 8 shows the two hypothesis molecule alignments in the derived final pocketmol along with the bound pose of celecoxib for reference. The alignments among the different inhibitors varied relatively little, with the differences between the training ligands and celecoxib in Fig. 8 exhibiting among the larger deviations.

**Fig. 9:**
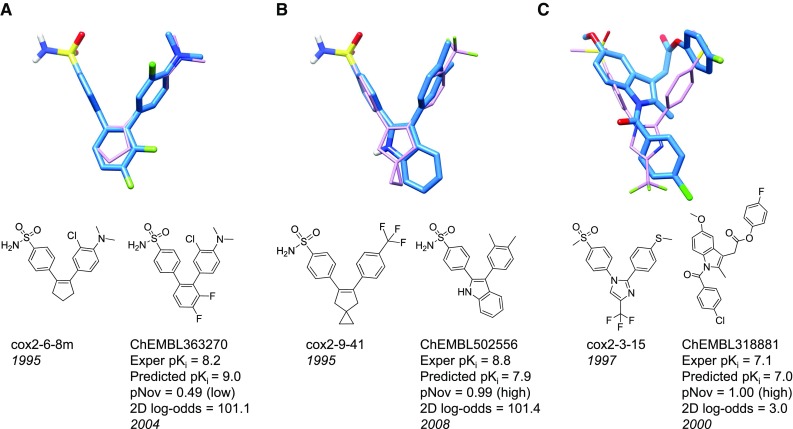
COX2 new ChEMBL molecules: molecule pairs comprised of a training molecule (*purple*) with maximum similarity to a new ChEMBL test molecule (*blue*) correctly predicted by QMOD to be an active COX2 ligand

Despite some central scaffold diversity within the COX2 dataset, there was very high 2D similarity of the COX2 test set to the training set (an average 2D log-odds of 135). Examination of the ChEMBL results revealed COX2 ligands with more structural variation, and these are shown in Fig. 9. Figure 9A shows an in-model prediction with a central phenyl ring, which, while being reasonably well predicted, was not terribly novel. Figure 9b shows an out-of-model prediction (pNov = 0.99) with a novel benzimidazole scaffold reported over a decade later than the nearest training ligand had been reported. If one considers predicted ChEMBL molecules with activity ${\ge} 7.0$, one begins to reveal much more novel scaffolds, as depicted in Fig. 9c. ChEMBL318881 had a significantly different structure from the training ligands, with a 2D log-odds of just 3.0. The corresponding 3D overlay with the most similar training ligand (which was a phenyl methyl sulfone) shows ChEMBL318881 with the 4-fluorophenyl ester group both non-planar and in the E conformation. Although this may not be the lowest energy state, this conformation of a 4-fluorophenyl alkyl ester is not unreasonable [41].

The ChEMBL assay data for COX2 had more variation than for the prior two targets, with average deviations between maximum and minimum reported activity values among those compounds with multiple values being close to 1.0 log units. For the 627 in-model compounds $\tau$ was 0.21 ($p < 0.01$, ties at 1.0 log units). There were 33 compounds whose predicted activity values were less than 5.0, which included some significant outliers. Considering only those ChEMBL compounds whose predicted activity was at least 5.0, $\tau$ was 0.30 ($p \ll 0.001$, ties at 1.0 log units).

It is possible to make use of multiple filters on new compounds, which create subsets on which predictions become more accurate. For example, considering the set with relatively low novelty (pNov $<$ 0.85) *and* relatively high confidence (pConf $\ge$ 0.35) produces 271 predictions, with a $\tau$ of 0.63 ($p \ll 0.001$, ties at 1.0 log units). Recall that the novelty measure considers whether a new ligand arranges itself so as to probe the binding pocket space in a manner that is well covered by the *union* of training molecules. The confidence measure addresses whether a *particular* training ligand is similar to the new ligand, as measured in their respective predicted poses.

### Thrombin

The Sutherland benchmark’s thrombin dataset contains ligands structurally similar to the potent inhibitor 3-TAPAP, which contains a central 3-amidino-phenyl-alanine scaffold with phenyl-sulfonyl and piperidine substituents [42]. Recall that Fig. 1 shows the initial alignment hypothesis (guided by structural information), the constraining effect of the methyl-benzamidine fragment, and the optimal final poses of the 59 training ligands. There were moieties which were relatively fixed in the pocket (e.g. the benzamidine) versus those with more flexibility (e.g. the substituted piperazine groups). The pocketmol probes formed three distinct regions corresponding to the thrombin pocket: acceptor probes mimicking the S1 pocket surrounding the benzamidine, and steric and donor probes representing both the S2 pocket around the substituted piperazines and the S4 pocket around the arylsulfonyl groups.

**Table 4:** ChEMBL datasets and screening utility of the ACHE, BZR, COX2, and thrombin QMOD models

	N molecules			N Positives			Winners			TP%	FP%	Enrichment
	ChEMBL	Sim	In-model	ChEMBL	Sim	In-model	N	Avg $\hbox {pK}_i$	N Pos	ChEMBL (Sim)	ZINC	TP/FP
ACHE	2454	342	162	493	86	57	28	7.6	18	3.7 (20.9)	0.0129	283
BZR	1158	843	129	309	234	30	34	7.8	24	7.8 (10.3)	0.9026	9
COX2	2322	1283	627	351	282	191	156	6.9	57	16.2 (20.2)	0.0064	2520
THR	3097	804	219	1069	251	81	31	7.6	19	1.8 (7.6)	<0.0064	>276

**Fig. 10:**
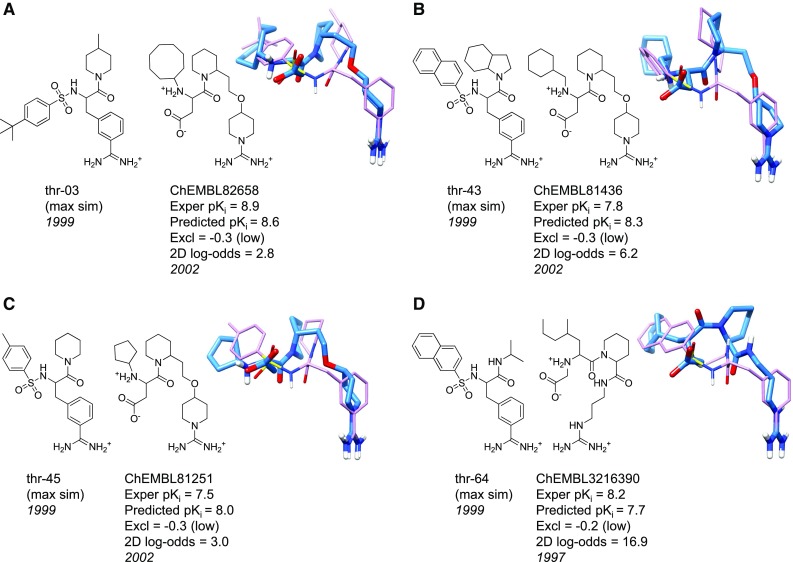
Thrombin new ChEMBL molecules: molecule pairs comprised of a training molecule (*purple*) with maximum similarity to a new ChEMBL test molecule (*blue*), each correctly predicted to be an active thrombin ligand

Screening ChEMBL compounds using the thrombin QMOD model provided an interesting difference from the other ChEMBL screens. Because the benchmark training set had little structural diversity (just a single central scaffold), *all* of the 804 ChEMBL thrombin ligands scored using the QMOD model were nominally out-of-model (pNov $\ge$ 0.85), and a number of molecules had substantial exclusion protrusions as well. For these reasons, the definition of in-model utilized raw values for similarity and exclusion penetration (similarity $>$0.70 and exclusion penalty $>-0.4$), which identified 219 molecules. Figure 10 shows four ChEMBL inhibitors predicted by QMOD to be winners. For each of the four examples, the predicted $\hbox {pK}_i$ was within 0.5 log units. The 2D similarity to the training sets of the ChEMBL molecules (2D log-odds range of 3.0–16.9) was significantly lower than that seen in the Sutherland benchmark’s thrombin test set (mean 2D log-odds = 95).

The ligand shown in Fig. 10a was perhaps the most interesting (the two in Fig. 10b, c were variations). It was predicted to have higher activity than any training ligand (whose maximum $\hbox {pK}_i$ was 8.5), and its experimental activity exceeded that of all training ligands. Instead of benzamidine in the S1 specificity pocket, a 1-amidinopiperadine is present. The linker to the S4 pocket was completely different than that seen in the training series. Also, the rank order among the three amidinopiperadines was predicted correctly, with the cyclooctane filling the S4 pocket more effectively than the cyclohexane or cyclopentane. Changes in the amine substituents created minor variations in the predicted bound poses, but all made similar interactions to the predicted binding pocket.

The ligand shown in Fig. 10d was one of just a few among all of the ChEMBL ligands for the four targets that was reported before the ligands used in the respective training sets. The inhibitor is inogatran, whose bound structure (PDB code 1K21) was among the five structures chosen for alignment hypothesis guidance, so its alignment with respect to the derived pocketmol was known. Recall that the structural guidance aspect of the model induction procedure *only* affected the initial alignment hypothesis, not the composition of the pocketmol or the final refined poses of the training ligands. The QMOD predicted pose deviated by 2.4 Å  RMS from the bound configuration of inogatran.

For a molecule with more than ten rotatable bonds, with such significant structural novelty, prediction of the activity and the binding mode at this level of accuracy should be able to support real-world application. Often, a single scaffold has been elaborated for structure-activity relationships, including some potent examples, but the scaffold may have liabilities that are not target-specific. In such cases, effective SAR transfer can support scaffold replacement. Here, given a training set with extremely limited structural variation, rank correlations on the in-model compounds was still highly statistically significant ($\tau =$ 0.26, $p \ll 0.001$), with predicted winners having an average experimental activity of 7.6. Predicted binding modes also qualitatively agreed with the well-understood behavior of thrombin inhibitors having cationic S1 recognition elements. In the thrombin case, rank correlation of all molecules, including out-of-model ones, was not statistically significant, owing to the narrowness of the training set compared with the diverse ChEMBL compounds.

### ChEMBL screening statistical analysis

Results for ACHE, BZR, COX2, and thrombin show how QMOD pocketmols can be used to predict the activities and poses of new, structurally diverse, molecules. This is possible because the QMOD model itself defines the optimal pose of a new ligand as the pose that best fits the model, and because the conformation and alignment optimization procedure is fully automated. However, because models are trained on limited structure-activity data, the utility of model application in a screening capacity is enhanced by making use of quantitative filtering in order to constrain the space of molecules on which predictions are likely to be accurate.

The procedure employed here first made use of pure 3D similarity-based virtual screening, utilizing the initial QMOD alignment hypotheses to identify molecules with a baseline level of similarity to the known ligands. Next, the models were run, with the top-ranked pose families being considered as potentially valid predictions. In-model predictions were those whose QMOD pNov parameter was less than 0.85 (for ACHE, BZR, and COX2), or in the case of thrombin where raw similarity and the exclusion penalty values exceeded particular thresholds (0.7 and -0.4, respectively). Further, a set of decoy molecules from ZINC were utilized in identical procedures to estimate false positive rates.

The foregoing has described rank correlation results as well as highlighting particular chemical structures. Table 4 summarizes results with respect to the numbers of compounds within each screening and filtering stage, as well as the estimates of true and false positive rates. The first group of three columns indicate, respectively, the total number of filtered ChEMBL compounds (see “Methods, data, and computational protocols”), the number in the similarity-based subset of ChEMBL molecules, and the number of in-model molecules. The next set of three columns indicates the number of positives (those compounds with experimental $\hbox {pIC}_{50}$ or $\hbox {pK}_i \ge 7.5$) within each of the three sets of molecules. The next trio of columns characterizes the nominal winners (in-model molecules with predicted activity $\ge$ 7.5) according to their total number, average experimental activity, and the total number of true positives among the winners.

The final three of columns in Table 4 characterizes the true positive rate, the estimated false positive rate, and the enrichment ratio. TP percentage is simply the percentage of total positives in the ChEMBL data set predicted to be winners by QMOD (the parenthetical number considers the true positives within the similarity-screened subset). The false positive rate was the percentage of decoys predicted to be winners. Estimated enrichment is the TP% divided by the FP%.

In all cases, the average experimental $\hbox {pK}_i$ for molecules predicted by QMOD to be winners was high, corresponding to 10–100 nM IC$_{50}$ or K$_i$. For three targets (ACHE, COX2, and thrombin), enrichment rates were very high, suggesting sufficiently specific predictions to be useful in prioritizing large sets of molecular candidates. A surprising outlier within these results was the estimated false positive rate for the BZR QMOD model, which was 100 times greater than the average for the other three targets. Due to this much higher rate, the computed enrichment was modest, but the true positive recovery rate was consistent with the other three targets. True positive rates for the full ChEMBL sets ranged from 2 to 16 %. The corresponding rates from among the subset that passed the similarity screen ranged from 8 to 21 %.

**Fig. 11:**
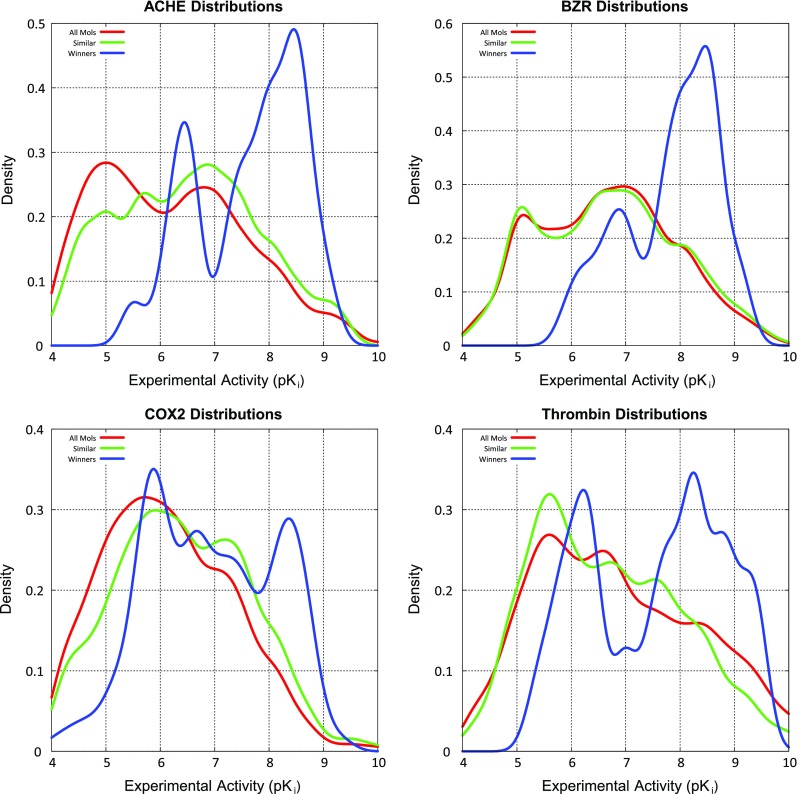
Distributions of experimental activity values for sets of ChEMBL compounds for four targets

The utility of a method for identification of potent new scaffolds depends on the extent to which nominal predicted winners have a large fraction of active molecules. Figure 11 shows the experimental activity distributions for the large ChEMBL dataset (red curve), the similarity-screened subset (green curve), and the set predicted to be winners by QMOD (blue curve). Not surprisingly, the similarity-based subset was not enriched for highly active molecules for any of the four targets. As with docking-based virtual screening, 3D similarity screening can be effective in identifying novel ligands that share specific binding with target ligands, but such methods are not directly useful for activity prediction. In contrast, the molecules predicted by QMOD to be winners were significantly enriched in active molecules and depleted in inactive ones ($p \ll 0.01$ by Kolmogorov–Smirnov).

### ACE and thermolysin

Angiotensin-converting enzyme and thermolysin are zinc metalloproteases with similar binding pockets, and accordingly these enzymes share a subset of inhibitors, albeit with different activities. Two ACE inhibitor drugs, enalapril and lisinopril, resulted from rational drug design projects based on thermolysin inhibitors [43–46]. Relevant to the datasets used in this work, SAR and crystallographic studies of ACE and thermolysin inhibitors revealed a minimum set of ligand moieties desirable for inhibition: (1) a zinc-chelating group such as a phosphonate, carboxylate, thiolate, or hydroxamate, (2) a carbonyl oxygen that hydrogen bonds to an active site residue, and (3) for ACE, a carboxyl group for ionic bonding to a positively charged residue of the enzyme [23]. In our preparation of these datasets, the zinc-coordinating groups were deprotonated to the charged forms known to be the enzyme-bound states.

All aspects of QMOD model induction and testing were similar for the two targets (see Tables 2, 3) (including rank correlation, average errors, and statistical significance for both training and testing on the Sutherland benchmark). Results for these two targets was numerically worse in terms of absolute errors than for the other targets, which is perhaps not surprising given that typical numbers of rotatable bonds for these largely peptide-like inhibitors often exceeded 15. Such extreme flexibility increases the burden on model induction and convergence as well as on the optimization of poses for new ligands. Rank-correlation results were consistent with the other targets. In the interest of space, this discussion will focus on ACE alone, as all observations hold equally for thermolysin.

**Fig. 12:**
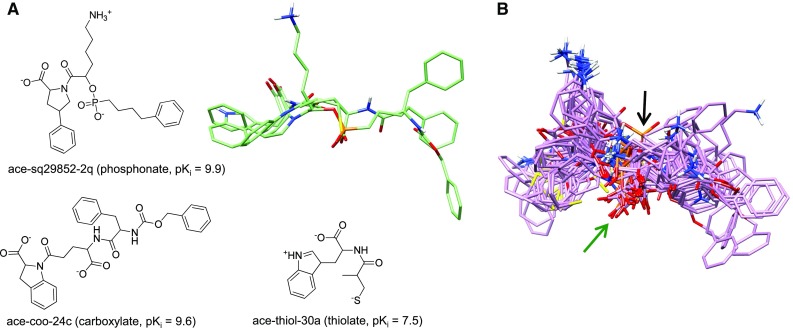
ACE QMOD model: **a** 2D structures and alignment hypothesis (*purple*), and **b** optimal final poses of the set ACE training ligands (*purple*)

Figure 12a shows the structures of the ACE hypothesis ligands and 3D structures for the ACE hypothesis and final optimal training poses. The final training poses showed some movement in the zinc-chelating groups, reflecting the known structural observation that zinc-chelating moieties have different preferred geometries [23]. Most of the zinc-chelating groups were correctly co-localized in the final optimal ligand poses (Fig. 12b, green arrow), but a few were misoriented, including a phosphonate group (black arrow).

The manual alignment procedure used previously for the Sutherland benchmark ACE ligands, described in [23], included constraints to superimpose the terminal carboxylate, the amide carbonyl, and the zinc ligand of each molecule. These alignments often resulted in incompatible chelation geometries, both within-class and between-class in terms of zinc chelation group. Nevertheless, the reports of 3D QSAR performance for the fixed alignment methods [8, 9] (see Table 3) were slightly better than those for QMOD. Interestingly, performance for 2D and 2.5D PLS-based QSAR was also reported [8], and ACE was one of only two targets where these methods performed well and equivalently to the 3D approaches. The ACE dataset appears to be one where it is possible to get right answer for the “wrong” reason using simple regression methods, but it represents a difficult case for a physically realistic method, due to the size and flexibility of the ligands. In addition, for both ACE and thermolysin, very broad activity ranges (roughly spanning 8–9 log units) present greater difficulties for methods such as QMOD that are not regression-based. Additional constraints on ligand alignments (e.g. providing preferred chelation fragment placement) would likely improve both training convergence and performance on the test set.

### Dihydrofolate reductase (DHFR)

DHFR was the other target for which 2D and 2.5D methods performed competitively with 3D methods in previous work [8]. The DHFR dataset was composed of several structural families, including variants of both folate and methotrexate. Given that all methods performed well (including non-3D ones), perhaps the most interesting aspect of the QMOD results on this target was that the model reflected a fully automated protocol for deriving initial and final, physically realistic, poses for the ligands. The Sutherland benchmark’s manual alignment of the 237 training ligands involved a labor-intensive procedure, making use of three different crystallographic structural templates along with numerous choices for how to position each substituent of each, rather flexible, ligand.

**Fig. 13:**
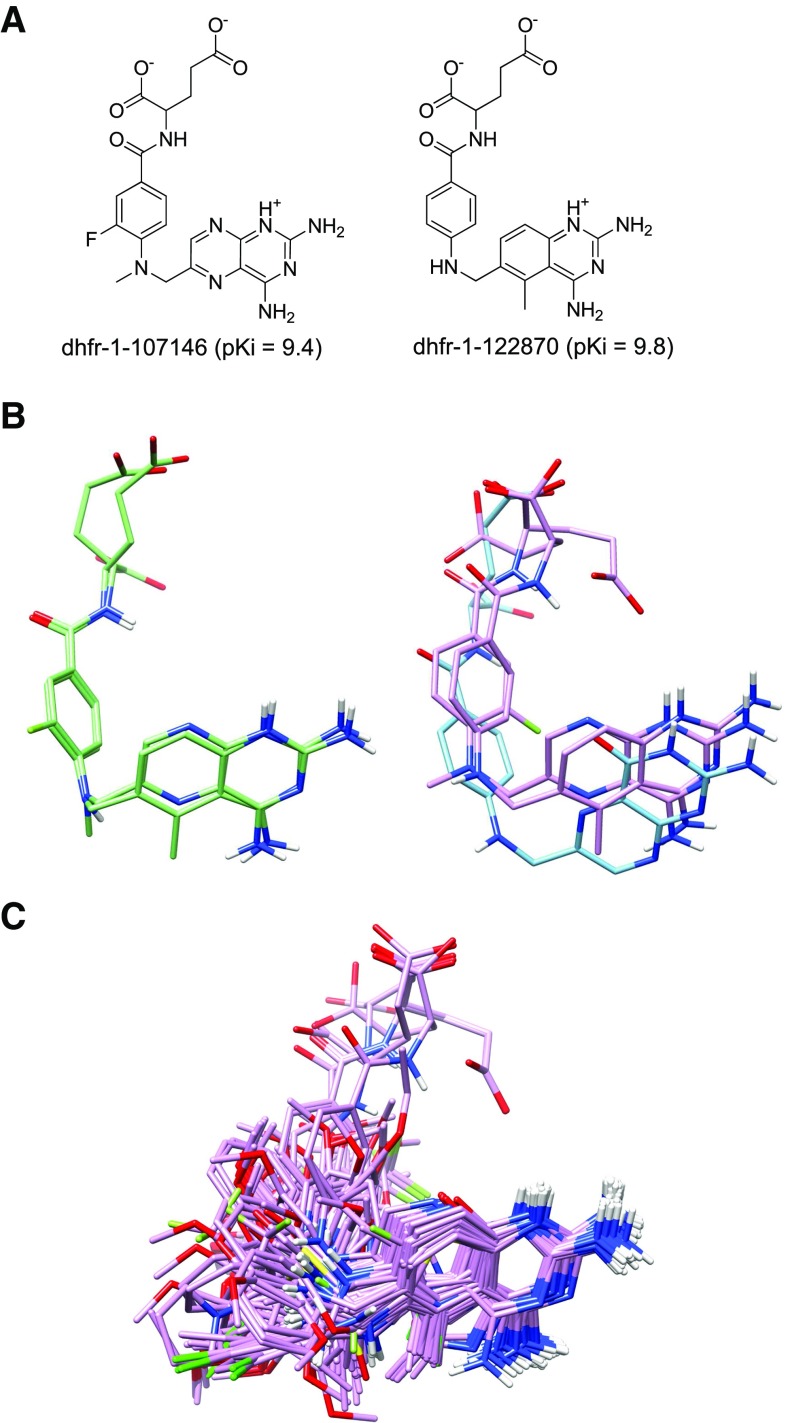
DHFR model: **a** 2D structures of training molecules, **b** structure-guided DHFR alignment hypothesis (*light green*) and final optimal poses for the two hypothesis molecules (*purple*) with native ligand folate (*cyan*) from structure 1DRF, and **c** optimal final poses representative training ligands (*purple*)

Figure 13 depicts the alignment results for the QMOD model, with the 2D ligands yielding a strongly congruent initial alignment (light green). Some movement during model induction occurred, as seen in Fig. 13b with the structure of bound folate shown for reference (cyan). The ring systems reflected the correct relative geometry based on experimental determinations. Representative poses of the optimal final poses of the training set is shown in Fig. 13c. Agnostic and automated generation of poses that have a physically meaningful relationship to reality is an advantage of the QMOD model building procedure, even in cases where there may be little or no numerical prediction advantages on a particular data set. As we have previously demonstrated [4, 7], as the structural diversity of new ligands increases, the prediction quality for models that match true bound ligand poses becomes much better than for models with poor agreement with the true protein–ligand interactions.

### Glycogen phosphorylase B

Glycogen phosphorylase catalyzes the release of glucose-1-phosphate from glycogen, and at least four distinct binding sites of this enzyme have been exploited as targets for type-2 diabetes therapies [47]. One of the earliest attempts employed glucose-analog catalytic site inhibitors [48], and glucose-analog dataset employed here contains inhibitors from that work [8, 25, 48, 49]. More recent studies have focused on GPB inhibitors that bind the AMP allosteric site with good potency (effective at nanomolar-level concentrations) [50].

Given that the majority of the GPB inhibitors in the dataset used here had $\hbox {pK}_i < 3.0$ (millimolar or worse effective concentrations), we did not consider this a relevant dataset from the perspective of drug discovery. Nonetheless, it was subject to the same procedures as with the other targets, and results were very similar to those reported for previous methods (see Tables 2, 3). Alignments for the ligands (both initial and optimized final poses) were unsurprising.

## Conclusions

We have reported results for the largest and most diverse public data set on which the QMOD method has been applied. The Sutherland benchmark is a challenging set for QSAR methods by design, in that the test compounds were identified in order to present extrapolative questions rather than interpolative ones. However, given the limitations of the most widely used 3D QSAR methods, the chemical series of the test ligands, in all cases, shared underlying scaffolds with at least some training molecules. Numerical test prediction performance among methods relying upon fixed alignments (CoMFA, CoMSIA, and CMF) and for QMOD was not significantly different overall, with inter-target variation dominating inter-method variation. The QMOD approach exhibited some weakness on highly flexible peptidic ligands (e.g. those of ACE and thermolysin), but showed strength in the particular case (BZR) where the automated alignment procedure produced a very different inter-scaffold correspondence than that assumed by the other methods.

Four algorithmic enhancements contributed to the ability of QMOD to yield convergent models and to provide interpretable results on diverse data derived from ChEMBL: 1) the complex optimization procedure for identifying initial pocket configurations has been engineered to be deterministic and focused upon finding parsimonious solutions; 2) the envelope of space explored by training ligands is now explicitly characterized and provides a soft boundary into which new ligands are encouraged to fit; 3) predicted pose families are produced and are ranked probabilistically, taking into account whether a pose looks like an outlier with respect to what is known; and 4) model building can be influenced using knowledge of binding modes while still allowing for broad application of the resulting models.

Application of the resulting models to large and diverse ligand sets from ChEMBL for four targets demonstrated four important features. First, fully automatic application of the models to predict activity and bound poses for structurally novel molecules was possible. Second, use of probabilistically normalized quality criteria to define a subset of molecular space was quantitatively useful in identifying predictions most likely to be accurate. Third, in all four cases, highly active molecules were identified with novel scaffolds, and in three of these cases, estimated enrichment rates were very high. Fourth, where such scaffolds were identified, their predicted poses were either clearly close to correct or presented plausible correspondence to the training ligands.

The choice to make use of a 2D QSAR method, a fixed-alignment 3D method, or a dynamic-alignment 3D QSAR method such as QMOD depends on what is required from the resulting models. QSAR methods that are 2D tend to be extremely fast, can often provide interpolative predictions that are quite accurate, and are not subject to any noise from pose optimization (or many aspects of ligand preparation). When data are plentiful and interpolation is valuable, application of such methods makes sense. Even in more complex cases, use of such methods can provide baseline performance estimates, as had been done previously for the data sets described here, and as we have done previously [6–8]. Methods requiring manual 3D alignment can be useful to go beyond what is possible with 2D methods to achieve a degree of extrapolation, as was shown in the work by Sutherland et al. in the work that described the data sets under study here [8]. However, there are practical challenges in constructing complex alignments and limitations in their breadth of application on new molecules. Further, in cases like the $\hbox {GABA}_{A}\hbox {R}$ benzodiazepine site, intuitive and easy-to-apply alignment rules may thwart the construction of models that generalize and predict well.

The QMOD method offers a quantitative means to address the ligand conformation and alignment selection process that respects physical constraints such as ligand energetics, can directly incorporate biophysical information, and mirrors the protein–ligand binding process in important ways. In cases where no information is known about the structure of a shared binding site for a set of ligands, an objective function based on 3D surface shape and electrostatics is used to produce initial alignments, which are then refined in the context of a physical model. QMOD models constructed with or without the use of structural knowledge using several dozen ligands from limited chemical series known at a particular time point can be used effectively to screen large parts of *future* chemical space to identify potent ligands with novel scaffolds.

Improvement to the QMOD method will be ongoing, with particular attention to speed of model induction and application, model selection questions when multiple convergent models exist, more careful treatment molecular charge distribution, and robustness in cases with very flexible ligands. In addition, systematic exploration of strategies and parameters for determination of the initial probe configuration will likely lead to improvements in performance. However, the results presented here suggest that the method is ready for broad, real-world application.

## References

[1] Langham JJ, Cleves AE, Spitzer R, Kirshner D, Jain AN (2009). Physical binding pocket induction for affinity prediction. J Med Chem.

[2] Jain A, Dietterich TG, Lathrop RH, Chapman D, Critchlow REJ, Bauer BE, Webster TA, Lozano-Perez T (1994). A shape-based machine learning tool for drug design. J Comput-Aided Mol Des.

[3] Jain A, Koile K, Chapman D (1994). Compass: Predicting biological activities from molecular surface properties. performance comparisons on a steroid benchmark. J Med Chem.

[4] Jain AN (2010). QMOD: physically meaningful QSAR. J Comput-Aided Mol Des.

[5] Jain A, Cleves A (2012). Does your model weigh the same as a duck?. J Comput-Aided Mol Des.

[6] Varela R, Walters W, Goldman B, Jain A (2012). Iterative refinement of a binding pocket model: active computational steering of lead optimization. J Med Chem.

[7] Varela R, Cleves A, Spitzer R, Jain A (2013). A structure-guided approach for protein pocket modeling and affinity prediction. J Comput-Aided Mol Des.

[8] Sutherland JJ, O’Brien LA, Weaver DF (2004). A comparison of methods for modeling quantitative structure–activity relationships. J Med Chem.

[9] Baskin II, Zhokhova NI (2013). The continuous molecular fields approach to building 3D-QSAR models. J Comput-Aided Mol Des.

[10] Cleves AE, Jain AN (2015). Chemical and protein structural basis for biological crosstalk between PPARa and COX enzymes. J Comput-Aided Mol Des.

[11] Kelley BP, Brown SP, Warren GL, Muchmore SW (2015). Posit: Flexible shape-guided docking for pose prediction. J Chem Inf Model.

[12] Jain AN (2000). Morphological similarity: a 3D molecular similarity method correlated with protein–ligand recognition. J Comput-Aided Mol Des.

[13] Cleves AE, Jain AN (2006). Robust ligand-based modeling of the biological targets of known drugs. J Med Chem.

[14] Yera ER, Cleves AE, Jain AN (2011). Chemical structural novelty: on-targets and off-targets. J Med Chem.

[15] Yera ER, Cleves AE, Jain AN (2014). Prediction of off-target drug effects through data fusion. Pac Symp Biocomput.

[16] Jain AN (2003). Surflex: fully automatic flexible molecular docking using a molecular similarity-based search engine. J Med Chem.

[17] Jain AN (2009). Effects of protein conformation in docking: improved pose prediction through protein pocket adaptation. J Comput-Aided Mol Des.

[18] Spitzer R, Jain AN (2012). Surflex-dock: docking benchmarks and real-world application. J Comput-Aided Mol Des.

[19] Golbraikh A, Bernard P, Chrétien JR (2000). Validation of protein-based alignment in 3D quantitative structure–activity relationships with CoMFA models. Eur J Med Chem.

[20] Maddalena DJ, Johnston GA (1995). Prediction of receptor properties and binding affinity of ligands to benzodiazepine/GABAA receptors using artificial neural networks. J Med Chem.

[21] Chavatte P, Yous S, Marot C, Baurin N, Lesieur D (2001). Three-dimensional quantitative structure–activity relationships of cyclo-oxygenase-2 (COX-2) inhibitors: a comparative molecular field analysis. J Med Chem.

[22] Böhm M, Stürzebecher J, Klebe G (1999). Three-dimensional quantitative structure–activity relationship analyses using comparative molecular field analysis and comparative molecular similarity indices analysis to elucidate selectivity differences of inhibitors binding to trypsin, thrombin, and factor Xa. J Med Chem.

[23] DePriest SA, Mayer D, Naylor CB, Marshall GR (1993). 3D-QSAR of angiotensin-converting enzyme and thermolysin inhibitors: a comparison of CoMFA models based on deduced and experimentally determined active site geometries. J Am Chem Soc.

[24] Sutherland JJ, Weaver DF (2004). Three-dimensional quantitative structure–activity and structure–selectivity relationships of dihydrofolate reductase inhibitors. J Comput-Aided Mol Des.

[25] Gohlke H, Klebe G (2002). DrugScore meets CoMFA: adaptation of fields for molecular comparison (AFMoC) or how to tailor knowledge-based pair-potentials to a particular protein. J Med Chem.

[26] Klebe G, Abraham U, Mietzner T (1994). Molecular similarity indices in a comparative analysis (CoMSIA) of drug molecules to correlate and predict their biological activity. J Med Chem.

[27] Thomas PD, Dill KA (1996). Statistical potentials extracted from protein structures: how accurate are they?. J Mol Biol.

[28] Kendall MG (1938). A new measure of rank correlation. Bibliometrika.

[29] Xu W, Hou Y, Hung Y, Zou Y (2013). A comparative analysis of Spearman’s rho and Kendall’s tau in normal and contaminated normal models. Signal Process.

[30] Cramer RD, Patterson DE, Bunce JD (1988). Comparative molecular field analysis (CoMFA). 1. Effect of shape on binding of steroids to carrier proteins. J Am Chem Soc.

[31] Wold S, Ruhe A, Wold H, Dunn W (1984). The collinearity problem in linear regression. The partial least squares (PLS) approach to generalized inverses. SIAM J Sci Stat Comput.

[32] Brown SP, Muchmore SW (2009). Large-scale application of high-throughput molecular mechanics with Poisson–Boltzmann surface area for routine physics-based scoring of protein–ligand complexes. J Med Chem.

[33] Jain A, Nicholls A (2008). Recommendations for evaluation of computational methods. J Comput-Aided Mol Des.

[34] Ordentlich A, Barak D, Kronman C, Ariel N, Segall Y, Velan B, Shafferman A (1998). Functional characteristics of the oxyanion hole in human acetylcholinesterase. J Biol Chem.

[35] Quinn DM, Feaster SR, Nair HK, Baker NA, Radic Z, Taylor P (2000). Delineation and decomposition of energies involved in quaternary ammonium binding in the active site of acetylcholinesterase. J Am Chem Soc.

[36] Bourne Y, Radić Z, Sulzenbacher G, Kim E, Taylor P, Marchot P (2006). Substrate and product trafficking through the active center gorge of acetylcholinesterase analyzed by crystallography and equilibrium binding. J Biol Chem.

[37] Bourne Y, Taylor P, Bougis PE, Marchot P (1999). Crystal structure of mouse acetylcholinesterase a peripheral site-occluding loop in a tetrameric assembly. J Biol Chem.

[38] Bergmann R, Kongsbak K, Sørensen PL, Sander T, Balle Balle, T (2013). A unified model of the GABAA receptor comprising agonist and benzodiazepine binding sites. PLoS One.

[39] Richter L, de Graaf C, Sieghart W, Varagic Z, Mörzinger M, de Esch IJ, Ecker GF, Ernst M (2012). Diazepam-bound $$\text{ GABA }_A$$ receptor models identify new benzodiazepine binding-site ligands. Nat Chem Biol.

[40] Hollinshead SP, Trudell ML, Skolnick P, Cook JM (1990). Structural requirements for agonist actions at the benzodiazepine receptor: studies with analogs of 6-(benzyloxy)-4-(methoxymethyl)-$$\beta $$-carboline-3-carboxylic acid ethyl ester. J Med Chem.

[41] Schaefer T, Penner GH (1987). The conformational properties of some phenyl esters. Molecular orbital and nuclear magnetic resonance studies. Can J Chem.

[42] Stürzebecher J, Prasa D, Hauptmann J, Vieweg H, Wikström P (1997). Synthesis and structure–activity relationships of potent thrombin inhibitors: piperazides of 3-amidinophenylalanine. J Med Chem.

[43] Patchett AA, Harris E, Tristram EW, Wyvratt MJ, Wu MT, Taub D, Peterson ER, Ikeler TJ, Broeke JT, Payne LG, Ondeyka DL, Thorsett ED, Greenlee WJ, Lohr NS, Hoffsommer RD, Joshua H, Ruyle WV, Rothrock JW, Aster SD, Maycock AL, Robinson FM, Hirschmann R, Sweet CS, Ulm EH, Gross DM, Vassil TC, Stone CA (1980). A new class of angiotensin-converting enzyme inhibitors. Nature.

[44] Hangauer DG, Monzingo AF, Matthews BW (1984). An interactive computer graphics study of thermolysin-catalyzed peptide cleavage and inhibition by n-carboxymethyl dipeptides. Biochemistry.

[45] Patchett AA, Cordes EH (1985). The design and properties of N-carboxyalkyldipeptide inhibitors of angiotensin converting enzyme.

[46] Natesh R, Schwager SL, Evans HR, Sturrock ED, Acharya KR (2004). Structural details on the binding of antihypertensive drugs captopril and enalaprilat to human testicular angiotensin i-converting enzyme. Biochemistry.

[47] Hadjiloi T, Tiraidis C, Chrysina ED, Leonidas DD, Oikonomakos NG, Tsipos P, Gimisis T (2006). Binding of oxalyl derivatives of $$\beta $$-D-glucopyranosylamine to muscle glycogen phosphorylase b. Bioorg Med Chem.

[48] Martin J, Veluraja K, Ross K, Johnson L, Fleet G, Ramsden N, Bruce I, Orchard M, Oikonomakos N (1991). Glucose analog inhibitors of glycogen phosphorylase: the design of potential drugs for diabetes. Biochemistry.

[49] Venkatarangan P, Hopfinger AJ (1999). Prediction of ligand–receptor binding thermodynamics by free energy force field three-dimensional quantitative structure-activity relationship analysis: Applications to a set of glucose analogue inhibitors of glycogen phosphorylase. J Med Chem.

[50] Kristiansen M, Andersen B, Iversen LF, Westergaard N (2004). Identification, synthesis, and characterization of new glycogen phosphorylase inhibitors binding to the allosteric AMP site. J Med Chem.

